# Metabolic and proteomic signatures differentiate inflammatory phenotypes from cancer and predict treatment response in patient sera

**DOI:** 10.1002/btm2.70029

**Published:** 2025-05-15

**Authors:** Gabriel Cutshaw, Elena V. Demidova, Philip Czyzewicz, Elizabeth Quam, Nicole Lorang, AL Warith AL Siyabi, Surinder Batra, Sanjeevani Arora, Rizia Bardhan

**Affiliations:** ^1^ Department of Chemical and Biological Engineering Iowa State University Ames Iowa USA; ^2^ Nanovaccine Institute Iowa State University Ames Iowa USA; ^3^ Cancer Prevention and Control Program Fox Chase Cancer Center Philadelphia Pennsylvania USA; ^4^ Department of Biochemistry and Molecular Biology University of Nebraska Medical Center Omaha Nebraska USA; ^5^ Cancer Epigenetics Institute Fox Chase Cancer Center Philadelphia Pennsylvania USA; ^6^ Department of Radiation Oncology Fox Chase Cancer Center Philadelphia Pennsylvania USA

**Keywords:** cancer patients, DNA damage response markers, metabolism, pancreatic cancer, Raman spectroscopy, rectal cancer, serum

## Abstract

Tumors shift their metabolic needs to enable uncontrolled proliferation. Therefore, metabolic assessment of cancer patient sera provides a significant opportunity to noninvasively monitor disease progression and enable mechanistic understanding of the pathways that lead to response. Here, we show Raman spectroscopy (RS), a highly sensitive and label‐free analytical tool, is effective in metabolic profiling across diverse cancer types in patient sera from both pancreatic ductal adenocarcinoma (PDAC) and locally advanced rectal cancer (LARC). We also combine metabolic data with proteomic signatures to predict treatment response. Our data show RS peaks successfully differentiate PDAC patients from healthy controls. Peaks associated with sugars, tyrosine, and DNA/RNA distinguish PDAC patients from chronic pancreatitis, an inflammatory condition that is notoriously difficult to discern from PDAC via current clinical approaches. Furthermore, our study is expanded to investigate response to chemoradiation therapy in LARC patient sera where at pre‐treatment multiple metabolites including glycine, carotenoids, and sugars are jointly correlated to the neoadjuvant rectal (NAR) score indicative of poor prognosis. Via classical univariate AUC–ROC analysis, several RS peaks were found to have an AUC>0.7, highlighting the potential of RS in identifying key metabolites for differentiating complete and poor responders of treatment. Gene set enrichment analysis revealed enrichment of metabolic, immune, and DDR‐related pathways associated with CRT response. Notably, RS‐derived metabolites were significantly correlated with multiple immune signaling proteins and DDR markers, suggesting these distinct analytes converge to reflect systemic changes within the tumor microenvironment. By integrating metabolic, proteomic, and DDR data, we identified pre‐treatment activation of galactose and glycerolipid metabolism, and post‐treatment engagement of cell cycle and p53 signaling pathways. Our findings show that RS, when integrated with complementary protein marker analysis, holds the potential to bridge the translational divide enabling a clinically relevant approach for both diagnosis and predicting response in patient samples.

**Translational Impact Statement**

This work demonstrates the translational impact of Raman spectral‐based metabolic analysis in cancer patient sera to differentiate cancer from inflammatory diseases that present very similar symptoms and are difficult to discern in the clinic. By integrating metabolite data with protein signatures and previously characterized DDR markers of treatment response, we identified coordinated metabolic–protein networks associated with chemoradiation outcomes in rectal cancer. Our minimally invasive clinically significant approaches using patient biofluids have the potential to minimize the need for repeated invasive biopsies for clinical decisions and predict a therapeutic response early to limit issues associated with treatment failures, such as unjustified toxicities and costs.

## 1 INTRODUCTION

Cancer cells have exquisite flexibility in rewiring their metabolic demand to support increased cell survival, differentiation, uncontrolled proliferation, and acquire treatment resistance. Tumor cells rely on core metabolic pathways such as glycolysis and the tricarboxylic acid (TCA) cycle to acquire nutrients and convert them to macromolecules and support their growth.^1^ Furthermore, oncometabolites including amino acids (AAs), nucleic acids, lipids and fatty acids (FAs), and antioxidants such as nicotinamide adenine dinucleotide phosphate (NADPH) are also associated with a pro‐tumorigenic effects.^2^ Indeed, this dysregulated cellular metabolism occurs at a much earlier timepoint before macroscopic changes in tumor burden are observed, thus serving as a key early biomarker of tumor progression. Therefore, metabolic reprogramming is now recognized as a significant early hallmark of cancer.^1^, ^3^ Recent advances in metabolite profiling highlight the relevance of biofluids such as blood, urine, and saliva as an alternative to tumor tissues to avoid repeat invasive biopsies in patients,^4^, ^5^ and the collection of these biofluids is often part of routine clinical procedures. Metabolic techniques that are sensitive, specific, rapid, and require minimal sample volumes are therefore imperative to enable accurate and early detection of cancer, differentiate cancer from inflammatory phenotypes that present similar symptoms, and identify markers of treatment response or resistance. In this work, we leverage Raman spectroscopy (RS), an optical approach that probes metabolic shifts in biological samples, to distinguish pancreatic ductal adenocarcinoma (PDAC) from pancreatitis, a non‐cancerous inflammatory disease, and examine treatment response in locally advanced rectal cancer (LARC). In RS, incident light interacts with molecules in a sample and results in molecular vibrations that give rise to a change in the frequency of the incident light.^6^ The resulting spectral characteristic is unique to each sample and corresponds to intrinsic metabolites. RS enables rapid, affordable, and clinically translatable biochemical analysis that has been implemented for disease detection and evaluation of treatment response in cancer and other disorders.^7^, ^8^, ^9^ Specifically, RS has advanced molecular profiling in cancer in sera,^10^, ^11^ in vitro models such as cells, spheroids, and organoids^12^, ^13^ and ex vivo systems including murine, canine,^14^ and patient tissues.^15^, ^16^ Here, we use RS to probe the serum metabolome of PDAC patients to understand the metabolic pathways that are activated in PDAC relative to chronic pancreatitis (CP) patients. Further, we also examine metabolic changes in LARC patient sera to distinguish poor responders from complete responders to adjuvant chemoradiation therapy. RS findings were correlated to serum signaling proteins, label‐free serum proteomics, and a subset of DNA damage response (DDR) markers in patient lymphocytes^17^ to understand the interplay between metabolism and immune function in facilitating a robust treatment response in LARC. We chose to explore multiple cancer types to demonstrate the broad utility of RS regardless of the cancer phenotype, as well as utilize the de‐identified patient samples that were available to us.

PDAC is a highly aggressive malignancy with a 5‐year survival rate < 10%.^18^ While PDAC is the most prevalent neoplasm of the pancreas, the lack of screening technologies for early detection presents a significant clinical challenge. Since distinct symptoms do not appear in PDAC until locally advanced disease or metastasis, PDAC is associated with a poor prognosis. Computed tomography (CT) remains the primary imaging tool for assessing cancer progression and treatment response. However, due to poor sensitivity, specificity, and low resolution, CT has high variability and low accuracy as a diagnostic tool,^19^ and often fails to distinguish cancerous lesions from non‐cancerous disease at the early stage.^20^ Blood‐based biomarker tests such as carbohydrate antigen 19‐9 (CA‐19‐9) tests are often used for the diagnosis of PDAC. While such tests are affordable and reduce the burden on patients and healthcare systems, the accuracy of CA‐19‐9 tests is low as this marker is often elevated in CP as well, leading to false positive diagnoses.^20^, ^21^ Differentiating PDAC from CP remains a clinical challenge due to similar symptoms and an inflammatory response in both diseases. Therefore, we leveraged the high sensitivity of RS to identify key metabolites and relevant metabolic pathways that are characteristic of PDAC to establish early markers of disease progression and ultimately improve patient survival.

LARC is also highly lethal, and colorectal cancers combined are the second leading cause of cancer death in the United States as of 2024.^22^ Treatment options are limited for LARC patients, where neo(adjuvant) chemoradiotherapy or systemic chemotherapy remains the current standard of care.^23^ Current clinical technologies cannot predict treatment response and fail to identify many patients that are likely to acquire resistance to initial therapies. Studies show that serum metabolites may serve as promising biomarkers of response in LARC patients.^24^ Yet how these metabolites correlate to protein markers and the resulting joint metabolic/protein pathways contributing to treatment resistance or response remains unexplored. Here we show that metabolites measured with RS in the LARC cohort are well correlated to neoadjuvant rectal (NAR) scoring specifically at pre‐treatment, suggesting metabolic analysis may be relevant in assessing who will respond to treatment before treatment is initiated.^25^ Furthermore, our findings show RS metabolites have moderate to strong correlation to both serum signaling proteins, proteins identified through proteomics, and DDR markers associated with response to DNA damaging chemoradiation therapy.^26^, ^27^ Our results show that the integration of metabolic + signaling proteins + DDR marker data together allows us to distinguish poor and complete responders to treatment, as well as identify key signaling pathways of response.

## 2 RESULTS AND DISCUSSION

Patient sera samples for this study were obtained from the Nebraska biobank for PDAC patients, CP patients, and control patients. We obtained *n* = 48 samples from patients without a cancer diagnosis (control group), *n* = 25 from patients with PDAC, and *n* = 20 from patients with CP. Of the 25 PDAC samples, 7 were found to display evidence of jaundice and were removed from the cohort, leaving a refined cohort of *n* = 18 PDAC samples, as jaundiced samples have been found to bias biomarker discovery for PDAC.^28^ These patients gave informed consent to donate these samples to the Nebraska Biobank for education and research purposes, but were not specifically enrolled in this study, so demographic and comorbidity information is unavailable. LARC patient sera samples were obtained from the Fox Chase Cancer Center (FCCC) from IRB study 22‐9925. For the analysis of serum cytokines, chemokines, and Raman metabolites, data were collected from a total of 99 patients. Among these, 16 patients had samples available from both before and after neoadjuvant chemoradiation therapy (nCRT). Additionally, serum samples were collected from 69 patients only after nCRT and from 14 patients only before nCRT. Overall, pre‐nCRT data were available for 30 patients, which included both cytokines and chemokines, as well as Raman metabolites. For these patients, both demographic and detailed treatment information was available and provided in Table 1. nCRT treatment details are provided in the methods section.

**TABLE 1:** LARC study patient demographics and nCRT surrogate endpoint, neoadjuvant rectal score.

Neoadjuvant rectal score and nCRT response groups	Average age (years)	# of male patients	# of female patients
Complete responder (NAR = <8)	60.6 ± 11.9	22	17
Partial responder (NAR = 8–14)	58.4 ± 11.1	4	6
Poor responder (NAR = >14)	51.1 ± 16.5	37	13

Metabolic changes in patient sera were measured with RS, where dilute serum was dried on a calcium fluoride substrate and samples were excited at 785 nm using a confocal RS microscope at ~40 mW power at the point of excitation using a 50X objective with a numerical aperture of 0.50. Each measurement consisted of 20 s exposure per point, and a total of 100 points was collected for each sample with a duration of ~35 min per sample. Raman spectra were processed using a custom Matlab processing pipeline (see “Methods”). The peaks of the processed spectra were then quantified for further analysis, where each peak is associated with key metabolites in serum. Tentative peak assignment is conducted by review of well‐established literature describing both the pure Raman spectra of potential metabolite candidates,^29^, ^30^, ^31^, ^32^, ^33^, ^34^, ^35^ as well as previous characterization of human blood components via RS.^7^, ^9^, ^36^ The tentative peak assignments used in analysis can be seen in Table 2.

**TABLE 2:** Tentative peak assignments for Raman spectral data, including the vibrational modes.

Wavenumber (cm^−1^)	Metabolic assignment	Vibrational modes	References
621	Phenylalanine	C–C twisting mode	29, 30, 31, 32
643	Proline	C–C twisting mode, skeletal stretching vibration	30, 31
671	DNA bases	C–S stretching mode	29, 30, 32
701	Cholesterol	Choline group, CH_2_ rocking, cholesterol ring deformation	30, 33, 34
719	Phosphatidylcholine (PC)/sphingomyelin	Symmetric stretch vibration of choline group	30, 33, 34
744	Thymine (DNA base)	Backbone vibrations, deformation of the ring	29, 30
757	Tryptophan	Symmetric breathing of tryptophan, *σ*(ring)	29, 30, 31
805	DNA/RNA	C′–O–P–O–C'3 phosphodiester stretching	30, 32
828	Tyrosine	Out‐of‐plane ring breathing	29, 30, 31
852	Sugars (glucose, glycerol)	C–O–C skeletal stretching	29, 30, 35
878	Glutamic acid	COOH deformation	29, 30, 31
898	Glycine	C–C stretching	29, 30, 31
938	Citric acid, Succinic acid	*υ* (OH‧‧‧O) out of plane wagging vibration of hydrogen bonds	29
957	Fatty acid	C–H bending	29, 33
992	Arginine	C–N stretching, C–C stretching	29, 31
1002	Phenylalanine	Symmetric in‐plane ring breathing	29, 30, 31
1011	Carbohydrates	C–O–C ring, C–O–H bending	29, 30, 35
1031	Phenylalanine	C–H in‐plane bending, ring in‐plane bending	29, 30, 31
1051	Glucose/glycerol	C–O stretching	29, 35
1063	Lipids	Chain C–C stretching	29, 30, 33
1082	Lipids	Chain C–C stretching	29, 30, 33
1103	Mannose	*σ*(CH_2_) twisting vibrations	30, 35
1126	Glucose	C–C stretching, C–O stretching, C–O–H in‐plane bending	29, 30, 35
1155	Carotenoids	C–C stretching, C–H stretching	29, 30, 34
1172	Saturated long‐chain fatty acids	C–C stretching	30, 33, 34
1207	Amino acids	NH_3_ asymmetric rocking	29, 31
1249	Amide III	Asymmetric O–P–O stretching	29, 30, 34
1257	Unsaturated lipids, fatty acids	CH_3_/CH_2_ twisting and wagging	30, 33, 34
1268	Unsaturated lipids, fatty acids	CH_3_/CH_2_ twisting and wagging	30, 33, 34
1314	Histidine	NH3+ asymmetric rocking, C–C–H stretching	29, 31
1338	Threonine	C–H deformation	29, 31
1448	Lipids and proteins	CH_2_ and CH_2_CH_3_ bending, scissoring, and deformation	30, 33, 34
1518	Carotenoids	C–C stretching and C=C stretching	29, 30, 34
1552	Tryptophan	Indole ring stretching, C=C stretching	29, 31
1575	DNA & NADH	Ring breathing modes	29, 30
1585	Phenylalanine	C–C bending	29, 30, 31
1605	Phenylalanine	C=O stretching, C=C in‐plane bending	29, 30, 31
1615	Tyrosine	C=C stretching	29, 30, 31
1657	Unsaturated lipids, PC, phosphatidylethanolamine	C=C stretching	30, 33, 34
1682	Amide I	C=O stretching	30

**FIGURE 1:**
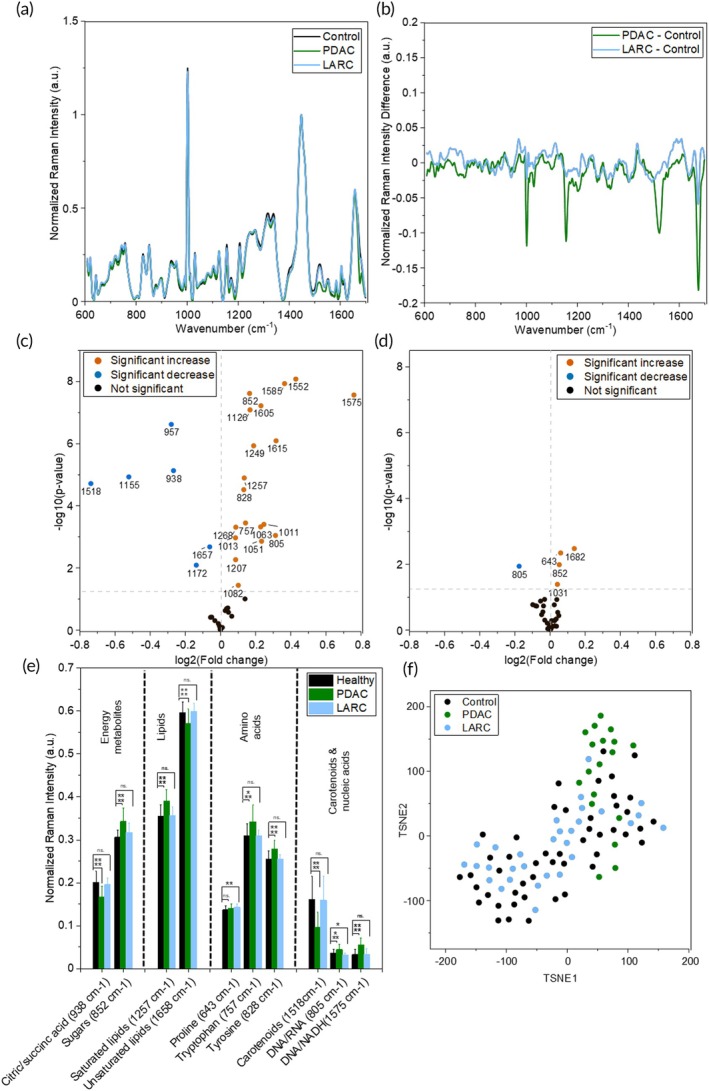
Raman spectral measurements of pancreatic ductal adenocarcinoma (PDAC) and locally advanced rectal cancer (LARC) patient sera. (a) Representative serum RS spectra for healthy, PDAC, and LARC patient samples. Representative spectra were selected from the centroid of the group's cluster from PCA. (b) Difference spectra were obtained by subtracting the healthy patient spectrum from the cancer patient spectrum. (c), (d) Volcano plots showing significance and fold change versus *n* = 48 healthy patient samples, for (c) *n* = 18 PDAC patient samples and (d) *n* = 30 LARC patient samples. Significance determined with a homoscedastic two‐tailed *t*‐test (*p* >0.05: Not significant, *p* <0.05: Significant). (e) Selected average RS peak values for *n* = 48 healthy patient samples, *n* = 18 PDAC patient samples, and *n* = 30 LARC patients. Significance determined with a homoscedastic two‐tailed *t*‐test (*p* >0.05: Not significant (ns.), *p* <0.05: *, *p* <0.01: **, *p* <0.001: ***, *p* <0.0001: ****). (f) PCA‐tSNE plot for TSNE1 and TSNE2 for *n* = 48 healthy patient samples, *n* = 18 PDAC patient samples, and *n* = 30 LARC patient samples.

Our initial goal was to leverage the high sensitivity of RS as a diagnostic approach and map the serum metabolome of patients who had PDAC or LARC and distinguish them from healthy non‐cancer controls. For this comparison, we used *n* = 48 healthy patient serum samples, *n* = 18 PDAC patient samples, and *n* = 30 serum samples from LARC patients prior to undergoing nCRT treatment. The Raman spectra of representative samples from each group (Figure 1a), and the corresponding difference spectra obtained by subtracting the control spectra from each representative cancer spectra (Figure 1b) show distinct serum metabolic profiles for PDAC and LARC. The Raman spectral features indicate fewer differences in serum metabolites of LARC patients compared to PDAC patients, suggesting that RS can accurately capture subtle metabolic differences among different cancer types. Using the mean peak values for each cohort, the specific peaks that differentiated either PDAC (Figures 1c and S1a) and LARC (Figures 1d and S1b) were analyzed. The volcano plot shows both relative changes in the mean value (fold change) and the statistical significance of that shift. For the PDAC cohort, 25 RS peaks showed significant differences versus the control patients. These include an increase in carbohydrates (1011 cm^−1^) and glucose (1126 cm^−1^), and the 852 cm^−1^ peak associated with sugars and especially disaccharides (Figures 1e and S1). The majority of pancreatic cancer patients suffer from the onset of diabetes mellitus, leading to hyperglycemia.^37^ PDAC has a high demand for glucose, so increased serum glucose levels may indicate tumor progression.^38^ The PDAC patient sera also showed a significant increase in peaks associated with long‐chain saturated FAs (1257 and 1268 cm^−1^) and a decrease in peaks associated with shorter FAs (957 cm^−1^) and unsaturated lipids (1657 cm^−1^). PDAC metabolic reprogramming has been found to upregulate enzymes associated with the synthesis of FAs and lipids, which promote cancer proliferation.^39^ De novo FA synthesis is linked to the TCA cycle and produced from citrate^40^; we observe a decrease in the citric acid associated 938 cm^−1^ peak in the PDAC patient cohort, suggesting consumption of citrate. In addition, multiple Raman peaks associated with AAs are also altered in the PDAC cohort that include increases in aromatic AAs such as phenylalanine (1031, 1585, and 1605 cm^−1^), tryptophan (757 and 1552 cm^−1^) and tyrosine (828and 1615 cm^−1^). AAs serve as an alternative fuel source for cancer cells to evolve and grow. Elevated AAs in the sera of PDAC patients may indicate protein breakdown and promote the malignant characteristic of tumors.^41^, ^42^ We also observed a significant downregulation in carotenoids (1155 and 1518 cm^−1^) as well as an increase in the 1575 cm^−1^ peak associated with DNA and NADH. A lower level of carotenoids is likely due to differences in dietary intake, although low levels of serum carotenoids are associated with high cancer risk.^43^ Our Raman spectral analysis supports that assessment of the serum metabolome is a useful approach for the rapid and accurate diagnosis of PDAC progression. In comparison to PDAC, we find only 5 significant peaks in LARC patient sera relative to the control group (Figure 1d, e). This included increases in the 852 cm^−1^ sugar peak and the 643 cm^−1^ peak associated with proline. Previous metabolomics investigation of rectal cancer patients found key carbohydrates increased versus healthy patients, as well as the upregulation of arginine and proline metabolism.^44^ Further, we applied unsupervised dimensionality reduction techniques including principal component analysis (PCA) (Figure S1c) and PCA‐t‐distributed stochastic neighbor embedding (tSNE) to the Raman spectral data to visualize (Figure 1f) the distinction between the cohorts. Both PCA and PCA‐tSNE show that serum metabolites do not strongly differentiate LARC patients from healthy controls, suggesting that metabolites from the tumor microenvironment (TME) are likely not released into the blood circulation of LARC patients for metabolic analysis. Both unsupervised methods show that PDAC samples differentiate and tightly cluster relative to healthy controls.

**FIGURE 2:**
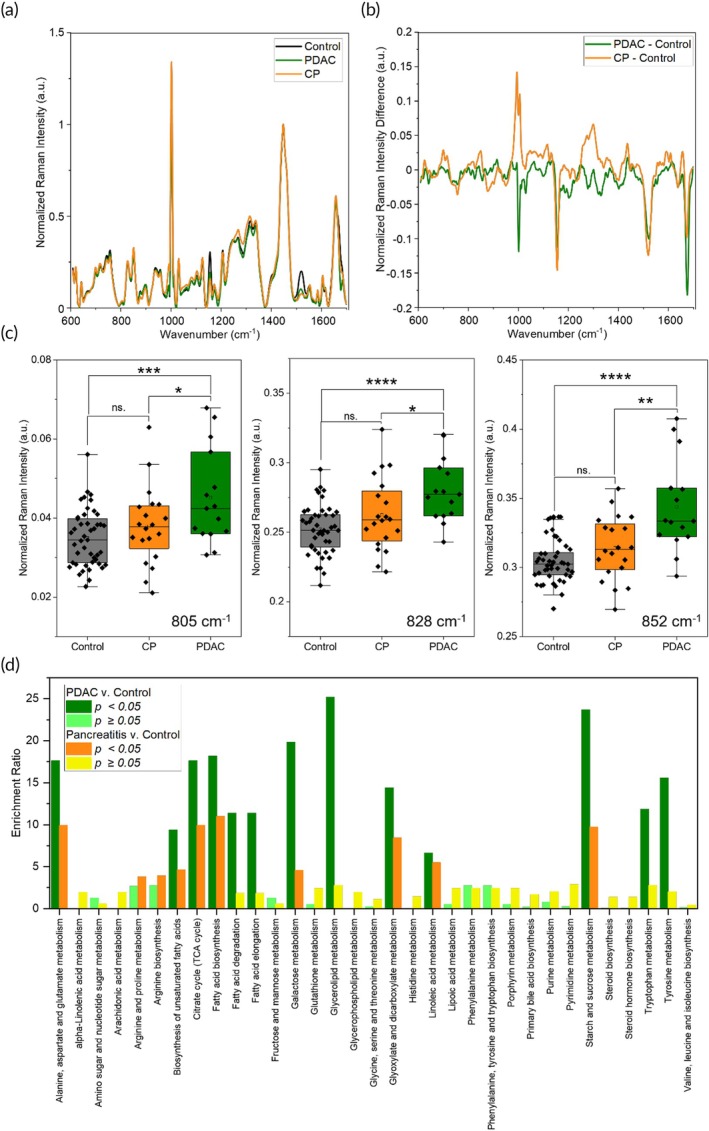
Raman spectral measurements of pancreatic ductal adenocarcinoma (PDAC) versus chronic pancreatitis (CP) patient sera. (a) Representative serum RS spectra for *n* = 48 healthy patient samples, *n* = 18 PDAC samples, and *n* = 20 CP samples. (b) Difference spectra were obtained by subtracting the healthy patient sample spectrum from the PDAC and CP sample spectra. (c) Selected RS metabolite peak values for *n* = 48 healthy patient samples, *n* = 18 PDAC patient samples, and *n* = 20 CP patient samples. Significance determined with a homoscedastic two‐tailed t‐test (*p* >0.05: Not significant (ns.), *p* <0.05: *, *p* <0.01: **, *p* <0.001: ***, *p* <0.0001: ****). (d) Quantitative metabolic pathway enrichment analysis for *n* = 18 PDAC patient samples and *n* = 20 CP patient samples versus *n* = 48 healthy patient samples. Tentative RS peak assignments used as proxies for metabolite with the KEGG pathway database. *P*‐values obtained from Metaboanalyst 6.0.

RS was then leveraged to distinguish PDAC patient samples from those with CP, a non‐cancerous inflammatory disorder that presents similar symptoms to early‐stage PDAC and is difficult to distinguish from PDAC clinically. We measured RS spectra of *n* = 20 anonymized CP patient sera, and the representative Raman spectra for CP, PDAC, and healthy control patients are shown in Figure 2a. Raman spectral features and the corresponding difference spectra (Figure 2b), obtained by subtracting PDAC and CP spectra from controls, indicate that PDAC and CP have many similarities but some differences as well (Figure S2) in their metabolic signature. For example, RS peaks associated with carotenoids (1155 and 1518 cm^−1^) showed similarities in the difference spectra between the PDAC and CP cohorts, whereas peaks associated with sugars (852 cm^−1^) and AAs (tyrosine, 828 cm^−1^) show differences. Comparisons of the peak values for CP and control patients (Figure S2) indicate 18 significant peaks that metabolically distinguish CP from healthy controls. Literature findings indicate that metabolic changes in glucose metabolism and lipid adsorption distinguish CP from healthy patients.^45^ But our measurements show that these pathways are also impacted in PDAC, which renders it difficult to differentiate PDAC and CP from serum metabolites. Unsupervised clustering analysis with PCA‐tSNE using the entire Raman spectra of samples showed limited differentiation between the PDAC and CP groups as well (Figure S3a). However, when specific RS peaks that distinguished the two cohorts were used with classical univariate receiver‐operator curve (ROC) analysis (Figure S3b), our results showed that these key peaks (Figure 2c) had predictive power to differentiate between the PDAC and CP groups. The peaks include the 852 cm^−1^ sugars peak (AUC = 0.76, 95% CI 0.61–0.91), the 828 cm^−1^ tyrosine peak (AUC = 0.72, 95% CI 0.54–0.88) and the 805 cm^−1^ DNA/RNA peak (AUC = 0.69, 95% CI 0.51–0.82).

We then interrogated if the RS metabolic peaks of PDAC and CP groups relative to the control patients would identify metabolic pathways that are enriched or suppressed in each group. We leveraged Metaboanalyst 6.0,^46^ a web‐based interface for quantitative enrichment analysis (Figure 2d) and found that PDAC is generally more metabolically dysregulated disorder relative to CP. Metabolites identifying as contributing to pathway enrichment can be seen in Table S1. This is reflected in higher alterations in metabolic pathways in PDAC patients such as those involving lipids and FAs including unsaturated FA biosynthesis, FA biosynthesis, FA degradation and elongation, and glycerolipid metabolism. PDAC reprograms lipogenesis to promote its growth and proliferation.^38^ Pathways associated with AAs such as alanine, aspartate and glutamate metabolism, tryptophan metabolism, and tyrosine metabolism were also impacted. We also see that the changes in tryptophan and tyrosine metabolisms are only significant in PDAC. As discussed earlier, in addition to glucose cancer cells rely on many AAs as a nutrient supply to promote “go or grow” phenomenon, that is, migrate to other sites and metastasize or proliferate primary tumors locally. Further pathways associated with energy metabolisms such as the citrate cycle, galactose metabolism, and starch and sucrose metabolism were also enriched in the PDAC group highlighting that serum metabolites are promising indicators for diagnosis of PDAC. A larger cohort study in future may identify additional metabolites that differentiate PDAC and CP.

Next, RS was leveraged to examine treatment response to chemoradiation therapy via serum metabolites in the LARC patient cohort. While metabolic enrichment analysis showed minimal differences comparing LARC and control patient groups (Figure S4), we conjectured that chemoradiation therapy in patients likely alters serum metabolites to distinguish responders and poor responders of clinical therapies. For the analysis of Raman metabolites, data were available from a total of 99 patients. Here, 30 pre‐treatment serum samples were compared to 85 serum samples from patients who had completed chemoradiation. These patients were further stratified through NAR scoring into complete response (NAR <8), partial response (14 > NAR >8), and poor response (NAR >14).^47^ For the pre‐treatment samples, 8 samples were from patients with a complete response endpoint, 11 from a partial response endpoint, and 11 from a poor response endpoint. For the post‐treatment samples, 34 were from complete responders, 7 were from partial responders, and 44 were from poor responders. Among these patient samples, we had 16 patients with matched pre‐ and post‐treatment samples; the patient demographics are provided in Table 1. To eliminate any unintentional bias of the dataset, only the pre‐treatment sample of each matched pair was used for longitudinal analysis.

**FIGURE 3:**
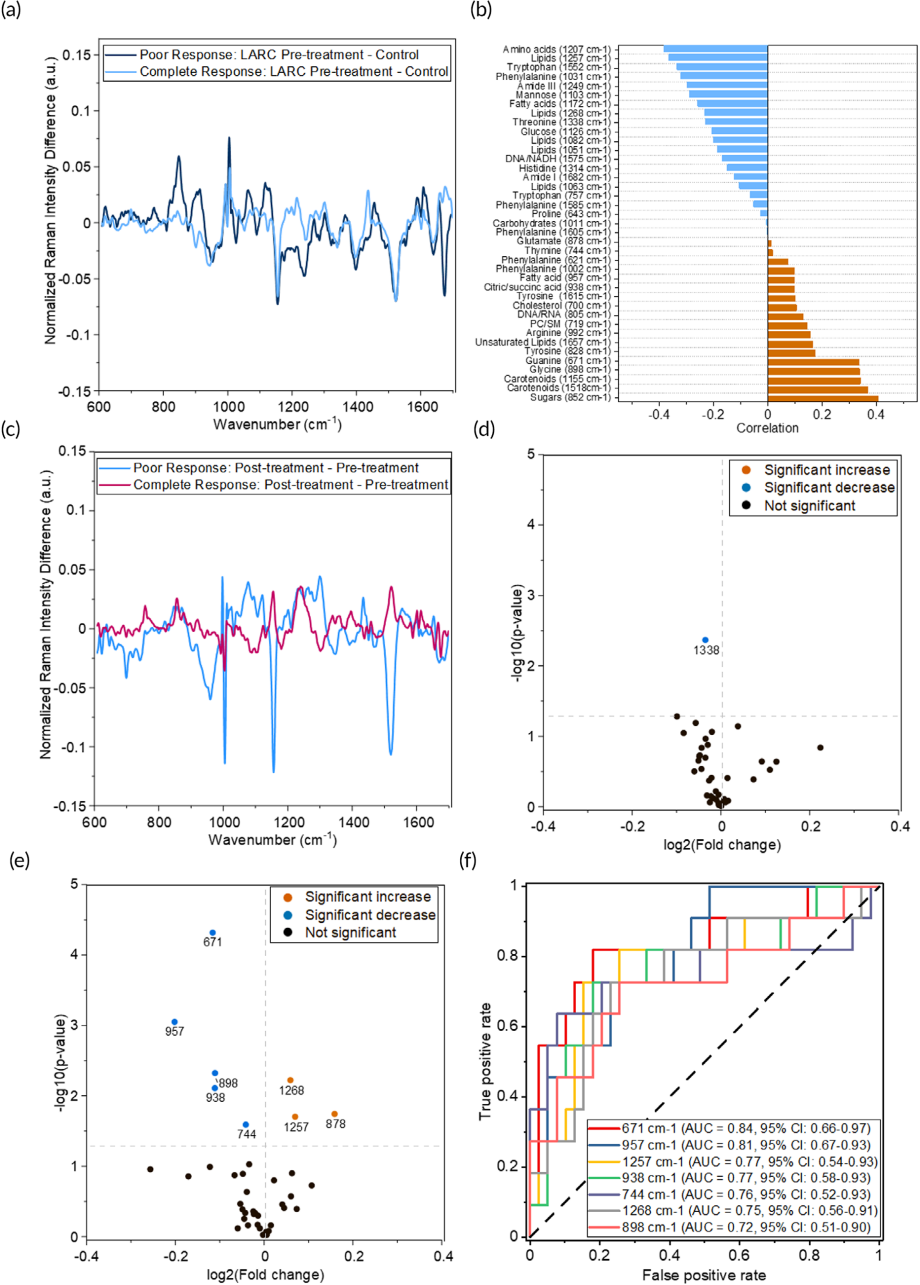
Raman spectral measurements of treatment response to chemoradiation therapy in locally advanced rectal cancer (LARC) patient sera. (a) Serum RS difference spectra for pre‐treatment LARC complete responder, and poor responder samples versus healthy patient samples. Difference spectra were obtained by subtracting the representative healthy patient spectrum from the representative LARC spectra. (b) Pearson's correlation of RS peaks against NAR score for *n* = 8 complete responder, *n* = 11 partial responde,r and *n* = 11 poor responder LARC patients. (c) Serum RS difference spectra between pre‐treatment and post‐treatment for LARC poor responder samples and complete responder samples. (d) Volcano plot showing significance and fold change for *n* = 11 pre‐treatment LARC poor responder samples and *n* = 39 post‐treatment LARC poor responder samples. Significance determined with a homoscedastic two‐tailed *t*‐test (*p* >0.05: Not significant., *p*<0.05: Significant). (e) Volcano plot showing significance and fold change for *n* = 19 pre‐treatment LARC completeand partial responder samples and *n* = 30 post‐treatment LARC completeand partial responder samples. (f) Classical AUC–ROC analysis for RS metabolite peaks for differentiation of *n* = 11 pre‐treatment LARC poor responder samples and *n* = 39 post‐treatment LARC poor responder samples.

The difference in Raman spectra pre‐treatment between poor responders and the healthy cohort, and complete responders and healthy (Figure 3a) as well as those at post‐treatment (Figure S5a) were compared. RS spectral differences highlighted more changes in many key metabolites at pre‐treatment relative to healthy. The correlation of these metabolites to the NAR score was performed via Pearson's correlation analysis at both pre‐treatment (Figure 3b) and post‐treatment (Figure S5b). At pre‐treatment, several metabolites had moderate joint (>0.3) correlation with the NAR score indicative of poor prognosis, which include glycine (898 cm^−1^), carotenoids (1155 and 1518 cm^−1^), and sugars (852 cm^−1^). The association of carotenoids with an increased NAR score was unexpected; although the benefits of carotenoids may be limited in rectal cancer.^48^ Glycine has been specifically identified as an indicator of poor prognosis in LARC tumors, as it contributes to tumor proliferation through one‐carbon mitochondrial metabolism.^49^ Several metabolites also had moderate inverse (<−0.3) correlation to NAR score including AAs (1207 cm^−1^) and saturated FAs (1257 cm^−1^) indicating that this broad class of metabolites play a complex role in the tumor microenvironment. The post‐treatment samples (Figure S5b) had minimal difference from the healthy controls, which is expected as metabolites that were reprogrammed during cancer progression could be reset post‐treatment reaching levels closer to the healthy cohort. Post‐treatment, no RS peaks showed moderate/strong correlation with the NAR score and there was minimal separation in PCA‐tSNE clustering (Figure S6a, b). To determine which metabolites could serve as prospective markers for poor response to treatment, we compared the pre‐treatment cohort of healthy controls relative to poor responders and those who were partial/complete responders (Figure S6c). We observe a decrease in the 805 cm^−1^ DNA/RNA peak which may be attributable to chemoradiation therapy that leads to DNA damage in both poor and complete responders. The 852 cm^−1^ sugar (glucose and glycerol) peak distinguishes the poor and complete responders and is elevated in poor responders indicating the tumors from these patients continue to rely on glycolysis as well as the glycerol 3‐phosphate shuttle that serves as a bridge between glycolysis and lipid metabolism.^50^

In addition to differentiating responders from poor responders both pre‐treatment and post‐treatment, we also investigated shifts in metabolites within the responder and poor responder patient groups. Difference spectra for representative samples at each timepoint (Figure 3c) showed metabolic changes in both poor and complete responders from pre‐ to post‐treatment. However, the Raman peak intensities had a higher degree of change for the poor responders of treatment. A volcano plot showing significance and fold change in the Raman peaks indicated that complete responders of treatment showed minimal change between time points (Figure 3d), but poor responders had 8 RS peaks that showed either significant increases or decreases after (unsuccessful) treatment (Figure 3e). Classical univariate AUC‐ROC analysis found that 7 out of these 8 RS peaks had an AUC> 0.7, suggesting that RS analysis has the potential to identify key metabolites for differentiating partial and complete responders versus poor responders of treatment in LARC (Figure 3f). The metabolites that increase post‐treatment include peaks associated with long‐chain saturated lipids (1257 and 1268 cm^−1^) and glutamate (878 cm^−1^) while peaks that decrease include peaks associated with DNA (671 and 744 cm^−1^), citric/succinic acid (938 cm^−1^), glycine (898 cm^−1^) and saturated FAs (957 cm^−1^). The shifts in citric/succinic acid and glutamate, and lipid dysregulation suggest that metabolic reprogramming of the tumors continues and intensifies when nCRT treatment is ineffective. Our findings suggest that RS analysis of patient sera may serve as an affordable tool for longitudinal monitoring of treatment response; a future study with a larger patient cohort and additional timepoints will be useful to determine the earliest timepoint of treatment prediction to ultimately enable effective therapies.

**FIGURE 4:**
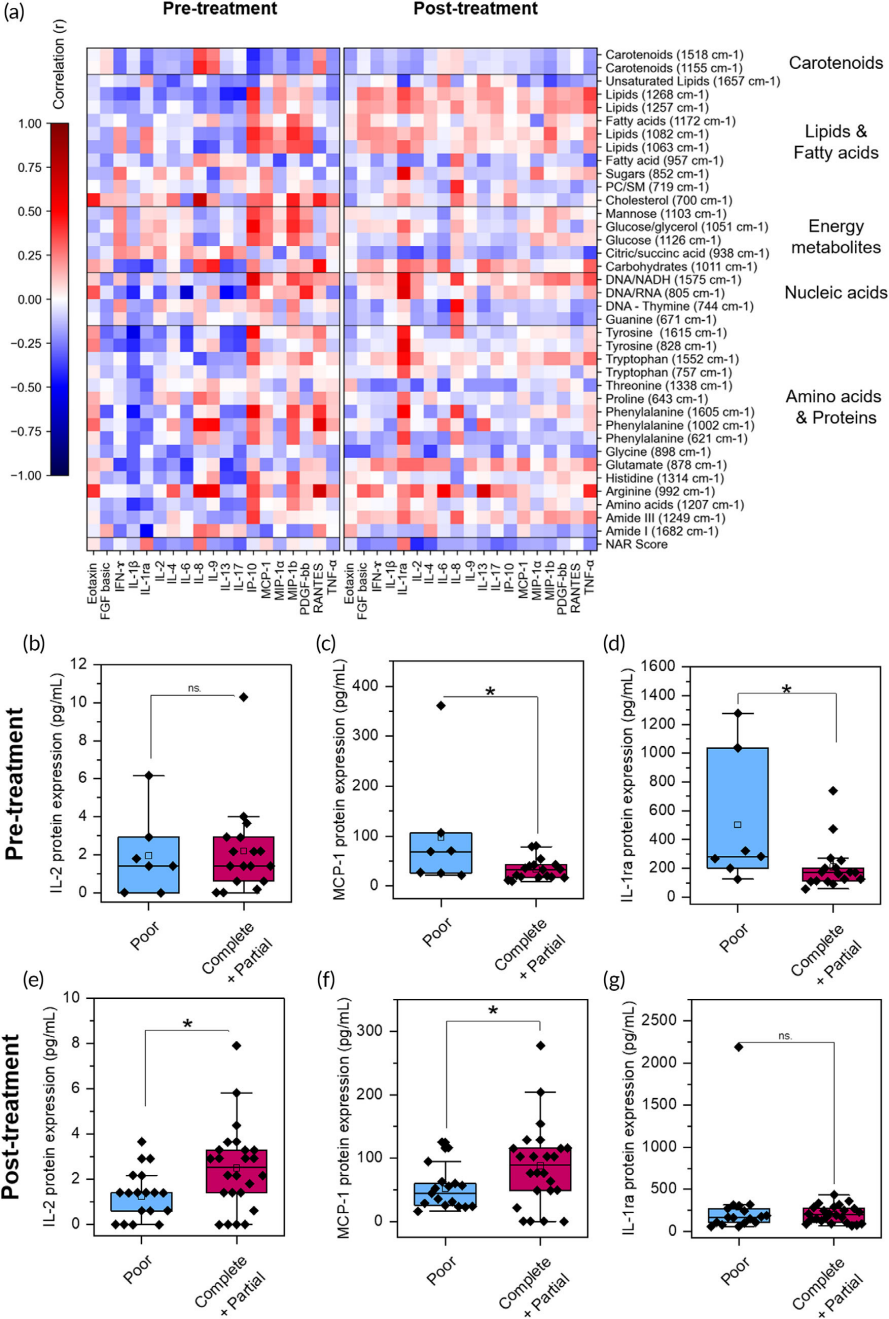
Correlation of Raman metabolites to serum signaling proteins and quantification of proteins in locally advanced rectal cancer (LARC) patient sera. (a) Correlation heatmap of RS peaks to serum signaling proteins that include chemokines and cytokines. For the pre‐treatment, correlations were conducted for *n* = 8 complete responders (NAR<8), *n* = 9 partial responders (8<NAR<14) and *n* = 7 poor responders (NAR > 14) LARC patients. For post‐treatment, correlations were conducted with *n* = 17 complete responders, *n* = 7 partial responders, and *n* = 17 poor responders LARC patients. (b)–(d) Protein concentration peak values of *n* = 19 complete or partial responders and *n* = 7 poor responders LARC pre‐treatment patients for (b) IL‐2, (c) MCP‐1 and (d) IL‐1ra levels. Significance determined with a homoscedastic two‐tailed *t*‐test (*p* >0.05: ns., *p* <0.05: *, *p* <0.01: **, *p* <0.001: ***, *p* <0.0001: ****). (e), (f) Normalized protein concentration peak values of *n* = 24 complete or partial responders and *n* = 17 poor responders LARC post‐treatment patients for (e) IL‐2, (f) MCP‐1, and (g) IL‐1ra levels.

Next, we studied key cytokines, chemokines, and growth factors in patient sera with enzyme linked immunosorbent assay (ELISA) with the ultimate goal of finding a correlation of serum metabolites to these cytokines and chemokines. Inflammation and immunomodulation are key to DNA damage in the TME and response to chemoradiation treatment, which can be assessed with serum cytokines/chemokines.^27^ For the pre‐treatment patients, we analyzed signaling proteins for *n* = 8 complete responders, *n* = 9 partial responders, and *n* = 7 poor responders. For the post‐treatment group, we analyzed *n* = 17 complete responders, *n* = 7 partial responders, and *n* = 17 poor responders. A Pearson's correlation analysis between metabolites, signaling proteins, and NAR score at both pre‐ and post‐treatment (Figure 4a) shows that the landscape of metabolite and cytokine correlations, and cytokines and NAR score correlations shift following treatment. Further annotated heatmaps with correlation values are provided in the supporting information (Figures S7 and S8). We find that IL‐17 shows a moderate inverse correlation (<−0.3) with NAR score at pre‐treatment, and IL‐2, IL‐4, and IL‐17 show moderate inverse correlations post‐treatment suggesting these cytokines are indicative of improved patient outcome. Several of these cytokines have paradoxical role in cancer. For example, IL‐17 has been shown to promote colorectal cancer growth,^51^ although meta‐analysis suggests that deletion of IL‐17 receptors in colorectal cancer can instead drive tumor growth.^52^ IL‐4 and IL‐10 have also been associated with radiosensitization,^53^ but meta‐analysis of rectal cancer datasets suggests that genes associated with IL‐4 signaling contribute to chemoradiotherapy resistance.^54^ We identified multiple cytokines that were well‐correlated with RS metabolites. At pre‐treatment, IL‐1*β* shows moderate inverse correlations with multipleAA peaks, including glutamic acid (878 cm^−1^), phenylalanine (621, 1585, and 1605 cm^−1^), tryptophan (757 and 1552 cm^−1^) and tyrosine (828 and 1615 cm^−1^). Studies have shown that radiation or chemotherapies increases IL‐1*β* production by tumor cells, which in turn recruits' neutrophils. These tumor‐associated neutrophils subsequently inhibit tumor growth.^55^ Therefore, an inverse correlation of IL‐1*β* to these key AAs is expected as cancer cells are addicted to glutamine, tryptophan catabolism, and other AA pathways to proliferate. Other notable correlations include that of IL‐8, which is strongly (>0.5) correlated with arginine (992 cm^−1^) and sugars (852 cm^−1^), and moderately correlated with carotenoids. IL‐8 promotes CRC proliferation and angiogenesis,^51^ and has been suggested as a possible biomarker for chemoradiation treatment in LARC.^56^ At post‐treatment, IL‐2 shows a joint correlation with arginine and an inverse correlation with threonine (1338 cm^−1^) and citric/succinic acid (938 cm^−1^). IL‐2 is considered to have antitumor effects in colorectal cancer,^51^ and murine studies have shown that IL‐2 can specifically facilitate radiotherapy response for rectal cancer.^57^ IL‐2, produced by activated T cells, enhances the antitumor therapeutic response by targeting effector T cells, memory T cells, and natural killer cells.^58^ A joint correlation of IL‐2 to arginine is not surprising as arginine is essential for normal immune function and immune surveillance, and it also destroys malignant cells.^59^ An inverse correlation of IL‐2 to TCA cycle metabolites (citric/succinic acid) may indicate a decrease in glycolysis since glucose metabolism is linked to the TCA cycle. Post‐treatment, IL‐1ra has a moderate to strong correlation with phenylalanine, tyrosine, tryptophan, DNA‐associated peaks, and several FA peaks. IL‐1ra is associated with poor prognosis in LARC and other cancer,^60^ and is correlated with both intramuscular and visceral fat in LARC patients.^61^ This suggests that metabolic dysregulations in cancer and those associated with poor treatment outcomes are also associated with other comorbidities (such as obesity). Furthermore, we also identified cytokines that differentiate the poor responders of treatment from the partial and complete responders. At pre‐treatment, IL‐1ra (AUC = 0.81, 95% CI 0.59–0.97) and MCP‐1 (AUC = 0.73, 95% CI 0.52–0.93) distinguished the two groups (Figure 4b) whereas at post‐treatment, IL‐2 (AUC = 0.72, 95% CI 0.56–0.86) differentiated the two groups (Figure 4c). MCP‐1 has a moderate negative correlation with Amide I (1682 cm^−1^), and moderate positive correlations with sugars (852 cm^−1^) and the 1082 cm^−1^ lipid peak at pre‐treatment. MCP‐1 expression in rectal cancer tumor cells is associated with recruitment of tumor‐associated macrophages, including in response to radiation therapy. Therefore, MCP‐1 upregulation at pre‐treatment may be an indicator of poor prognosis.^62^ Unsupervised differentiation of the samples (Figure S9) using RS + signaling protein data shows some separation between poor and complete responders post‐treatment with PCA.

**FIGURE 5:**
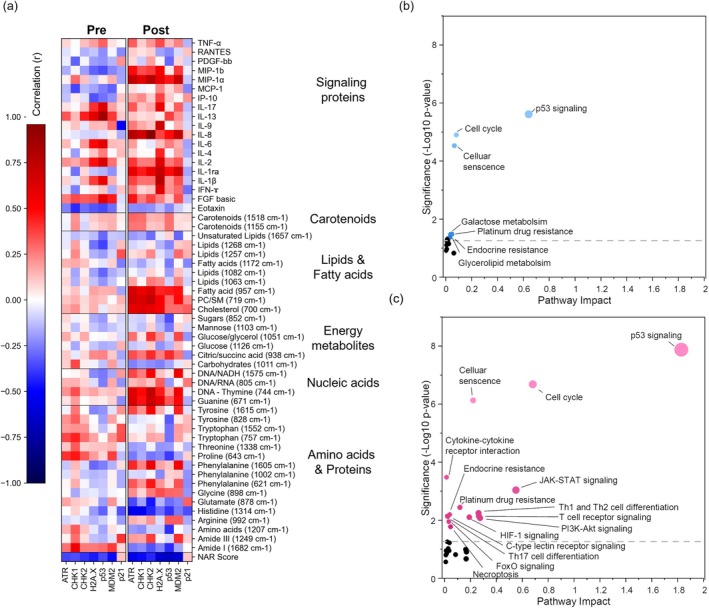
Correlation of Raman metabolites to DNA damage repair (DDR) markers and joint protein‐metabolite pathways in locally advanced rectal cancer (LARC) patient sera. (a) Correlation heatmap of DDR lymphocyte markers to RS metabolites and serum signaling proteins. For the pre‐treatment, correlations were conducted for *n* = 8 complete responders (NAR<8), *n* = 8 partial responders (8 < NAR < 14), and *n* = 7 poor responders (NAR > 14) LARC patients. For post‐treatment, correlations were conducted with *n* = 8 complete responders and *n* = 10 poor responders LARC patients. Joint protein and metabolite pathway analysis using combined RS metabolite + cytokine + lymphocyte DDR marker data of (b) pre‐treatment LARC patients comparing *n* = 8 complete responders and *n* = 7 poor responders, and (c) post‐treatment LARC patients comparing *n* = 8 complete responders and *n* = 10 poor responders.

To further understand the relationship between metabolites and immune response in treatment outcomes of LARC, we analyzed key protein markers associated with activated DDR signaling in the lymphocytes of selected LARC patients. For comparison of DDR markers, data were available from only 41 LARC patients.^17^ It is well‐established that genotoxic stress such as chemotherapy and radiation induce inflammatory cytokines and chemokines signaling in the tumor microenvironment.^63^ Furthermore, several markers of activated DDR signaling including in immune cells have been associated with increased response to chemoradiation therapy in LARC.^17^ Markers measured include ataxia telangiectasia and Rad3‐related protein (ATR), checkpoint kinases 1 and 2 (CHK1 and CHK2), mouse double minute 2 homolog (MDM2), H2A histone family member X (H2A.X) and CDK‐interacting protein 1 (p21) and tumor protein 53 (p53). These proteins play key roles in recognizing and facilitating the repair of DNA damage.^64^ Here, the correlation of DDR markers to Raman metabolites was conducted on a limited set, as only a small number of patient samples were available for the assay. For the pre‐treatment samples, analysis was conducted with *n* = 8 complete responders, *n* = 8 partial responders, and *n* = 7 poor responders LARC patients. For post‐treatment, analysis was conducted with *n* = 8 complete responders and *n* = 10 poor responders LARC patients. As shown before,^17^ and as the correlation heatmap shows (Figure 5a), the DDR signaling markers, measured in peripheral lymphocytes, are correlated with response to therapy. These correlations are moderate at pre‐treatment and moderate to strong at post‐treatment. Furthermore, an annotated heatmap with Pearson's correlation values is provided in the supporting information (Figures S10 and S11).

We also find interesting correlations of these DDR markers to cytokines and chemokines, which is discussed first, as well as to metabolites, which is discussed next. For example, MDM2 and p53 have a moderate to strong joint correlation to IL‐6 and IL‐13 pre‐treatment that significantly dampens post‐treatment. IL‐6 and IL‐13 are both associated with tumor growth, inflammation, and metastasis in colorectal cancer.^65^ The pre‐treatment correlation suggests that tumorigenesis progression leads to an increased inflammatory response that reduces post‐treatment. Conversely, MDM2 shows low correlation to MIP‐1*α* and *β* (also known as CCL3 and CCL4 respectively) at pre‐treatment, which changes to strong joint correlation post‐treatment. Activation of MDM2 signaling promotes CD8^+^ T‐cell survival, which suggests that IL‐6 and IL‐13 have pro‐tumorigenic effects in the samples tested while MIP‐1*α* and *β* have a therapeutic effect, promoting immune cell chemotaxis and accumulation.^66^ Similar correlations were observed between p53 and IL‐6, IL‐13, and MIP‐1*α* and *β*. p53 is a key signaling marker associated with T‐cell activation; p53 plays an active role in transitioning T cells from a quiescent to active state.^67^ p53 also showed joint correlation to IL‐2, which is produced by activated T cells, and to IL‐17, which is involved in T cell differentiation. DNA damage surveillance has been linked to memory T cell differentiation.^68^ DNA damage surveillance capacity is associated with the expression of H2A.X: our results show strong correlations between H2A.X and both IFN‐γ and IL‐2 at post‐treatment (Figure 5a). These correlations highlight that memory T cell differentiation leads to a durable immune response likely enabled by the proliferation of effector T cells.^69^ We see similar correlations with ATR and its downstream CHK1 and CHK2 proteins against these cytokines where ATR and the ATR‐CHK pathways are known to activate B lymphocyte development.^70^, ^71^

The DDR markers also show significant correlation to several RS metabolites. MDM2 and p53 have moderate‐to‐strong positive correlations with metabolites associated with DNA bases (671, 744 cm^−1^) and NADH (1575 cm^−1^). Nucleotide and NADH metabolism is essential to DNA damage repair and activates MDM2 and p53 pathways.^72^, ^73^ p53 and MDM2 also show joint correlation at post‐treatment to arginine (991 cm^−1^), some FAs (957 cm^−1^) and TCA cycle metabolites (938 cm^−1^). Since memory T cells rely on FA oxidation, and effector T cells are driven by glycolysis/TCA cycle, the correlation of p53 and MDM2 to these key metabolites supports that metabolic reprogramming is important for memory T cell differentiation and prolonged immune response.^74^ p53 also shows a moderate inverse correlation with several peaks associated with AAs, including proline (643 cm^−1^), tyrosine (828 cm^−1^), histidine (1314 cm^−1^) and generic AA peak (1207 cm^−1^). This may suggest an exhaustion phenotype associated with p53 activation: the lack of AA substrates can constrain T lymphocyte mTOR pathway activity, thereby suppressing the glycolytic effector metabolism.^75^ ATR and its downstream CHK1 and CHK2 proteins show strong joint correlations with cholesterol (700 cm^−1^), membrane lipids (719 cm^−1^) and the 957 cm^−1^ FA peak. Cross‐talk between ATR and FA synthesis has been previously reported, with saturated FAs suppressing ATR activation of p53.^76^ Production of membrane phospholipids is accomplished through FA synthesis pathways in T lymphocytes.^77^ However, not all FA peaks show correlations to ATR and its downstream proteins (such as 1172, 1268 cm^−1^) indicating the complex role of lipids and FAs in DDR signaling. Further study into differential lymphocyte FA metabolisms may elucidate these differences in response to nCRT.

The combined Raman metabolite data and serum cytokine and lymphocyte DDR marker data was used to assess the signaling pathways that are activated using a topology‐based approach with Metaboanalyst 6.0's joint pathway‐analysis module. Features matched to pathways can be seen in Tables S2 and S3. Unsurprisingly, the p53 signaling pathway is highly relevant both pre‐treatment (Figure 5b) and post‐treatment (Figure 5c) but the impact and significance of the pathway increases post‐treatment, supporting the role of p53 in therapeutic response. Furthermore, pathways associated with cell cycle regulation were also enriched with an increase in impact post‐treatment. This finding supports the correlation observed between individual DDR signals and prognosis based on NAR score (Figure 5a); this may indicate that the activation of cell cycle regulation pathways serves to predict response pre‐treatment and differentiates responders from poor responders post‐treatment. In addition, the JAK–STAT and PI3K‐AKT pathways are also significant post‐treatment, indicating that chemoradiation therapies likely impact these downstream signaling pathways in LARC patients. These two pathways are also relevant in lymphocyte signaling, where the JAK–STAT pathway regulates homeostasis in T cells, enabling immune response,^78^ and PI3K signaling is relevant in T cell trafficking and metabolism.^75^ As seen in Figure 3, metabolic pathways show greater differential enrichment at pre‐treatment, including galactose and glycerolipid metabolisms, which are also present in the joint metabolic and protein pathway (Figure 5b). Indeed, the combination of metabolic + signaling proteins + DDR marker data collectively allows us to differentiate poor and complete responders in PCA analysis (Figure S12) suggesting that integration of these distinct datasets is key to developing an accurate model for predicting response to chemoradiation treatment in patients. To assess the impact of the dataset combinations, we performed PCA‐support vector machine (SVM) analysis for different combinations. In each case, the dimensionality of the dataset was reduced to 2 for each feature set via PCA, and a linear kernel SVM was trained and evaluated using leave‐one‐out cross‐validation (Table S4), which shows integrated analysis of RS + Cytokine + DDR markers improves differentiation, thus improving prediction of treatment response.

To further understand the role of various proteins in treatment response in the LARC cohort, we profiled the patient serum proteome post‐treatment for the *n* = 8 complete responders and *n* = 10 poor responders. We used liquid chromatography–mass spectrometry (LC–MS) proteomics to assess digested serum peptides that identified 284 unique serum proteins. Combined with the 19 characterized signaling proteins and 6 characterized DNA damage markers in LARC from our prior work,^17^ we have a total of 309 identified proteins for downstream analysis. Summaries of the ontology of these proteins are provided in the SI (Figure S13).

**FIGURE 6:**
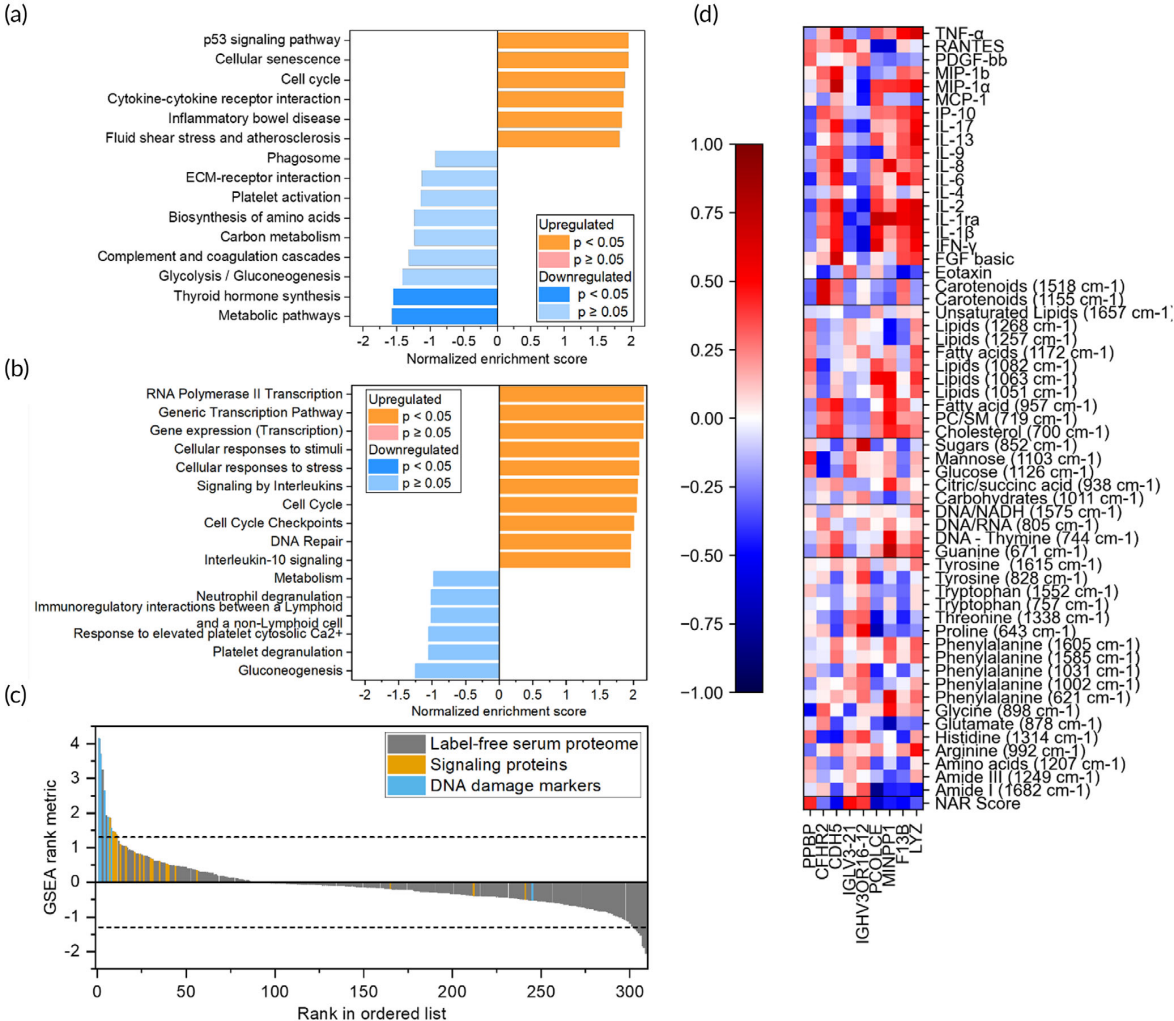
Serum proteomics analysis for differentiating complete and poor responders post chemoradiation treatment. (a), (b) Gene set analysis (GSEA) for signaling proteins, DNA damage markers, and label‐free serum proteomics for *n* = 8 complete responders and *n* = 10 poor responders post‐treatment. *P*‐values were reported by WebGestalt 2024. Analysis was completed using the (a) KEGG pathway database and (b) Reactome pathway database. (c) 309 identified and mapped proteins ranked and ordered with the GSEA rank metric. A positive metric indicates that the protein was upregulated in complete responders, and a negative metric indicates that it was downregulated in complete responders. Dashed lines demarcate the cut‐off for differential expression (|GSEA rank metric|>1.30). (d) Correlation heatmap of differentially expressed serum proteins to RS metabolites and serum signaling proteins. The differentially expressed proteins identified from LC–MS proteomics include platelet basic protein (PPBP), complement factor H related 2 (CFHR2), cadherin‐5 (CDH5), immunoglobulin lambda variable 3–21 (IGLV3‐21), immunoglobulin heavy variable 3/OR16‐12 (IGHV3OR16–12), procollagen C‐endopeptidase enhancer 1 (PCOLCE), multiple inositol polyphosphate phosphatase 1 (MINPP1), coagulation factor XIII (F13B) and lysozyme C (LYZ). Correlations were conducted with *n* = 8 complete responder and *n* = 10 poor responder LARC patients.

Gene set analysis (GSEA) was performed using the online WebGestalt 2024 platform^79^ to investigate both KEGG and Reactome reaction pathways (Figure 6a, b). Proteins that contributed to these pathways are shown in the SI (Tables S5 and S6). The distribution of identified proteins by GSEA rank metric is presented in Figure 6c. Through this expanded proteome, we show pathway analysis that includes significant differential enrichment of p53 signaling, cellular senescence, and cell cycle pathways all closely related to DDR pathways, as well as cytokine signaling. Furthermore, several pathways were also downregulated in complete responders such as thyroid hormone synthesis (*p* = 0.016) and metabolic pathways more broadly (*p* = 0.039). Hypothyroidism is associated with reduced rectal cancer risk as thyroid hormones can stimulate cell proliferation and angiogenesis in rectal cancer.^80^ In our study, pathways related to thyroid hormones are driven by changes in glutathione peroxidase 3 (GPX3, GSEA rank metric = −0.69) and thyroxine‐binding globulin (SERPINA7, GSEA rank metric = −0.68), although neither protein is differentially expressed individually. To perform correlation analysis, we normalized the abundance of this differentially enriched subset to the maximum across the patients and assessed correlations versus each patient's RS metabolite and signaling proteins. Pro‐platelet basic protein (PPBP) expression moderately correlated with NAR score (*r* = 0.43). PPBP has been identified as a hub gene in cytokine and chemokine signaling pathways in rectal cancer and CRC.^81^, ^82^ Our results show PPBP expression has inverse correlations with multiple cytokines and chemokines (IL‐2, IL‐6, IL‐13, IL‐17, IP‐10) and a joint correlation with PDGF‐bb (*r* = 0.31). The platelet to lymphocyte ratio is a good prognostic marker of inflammation and overall prognosis in rectal cancer patients.^83^ PPBP also shows correlations to metabolites, including a strong inverse correlation to glycine (898 cm^−1^). Studies show glycine reduces platelet aggregation and growth factor expression in pre‐clinical studies^84^, ^85^; however, the exact role of serum glycine levels in platelet factor expression needs further investigation. Our findings also show expression of cadherin‐5 (CDH5) or VE‐cadherin, which has a strong inverse correlation to NAR (*r* = −0.54). Cadherins are major drivers of metastatic tumor progression^86^; CDH5 in particular regulates endothelial barriers facilitating immune infiltration.^87^, ^88^ CDH5 also has moderate to strong joint correlation to multiple pro‐immunity cytokines (IFN‐*γ*, IL‐1*β*, IL‐2, MIP‐1*α*, and MIP‐1b), indicating that immune response is likely key to nCRT therapeutic response in LARC. CDH5 also has moderate joint correlation to membrane lipids (cholesterol 700 cm^−1^, PC/SM 719 cm^−1^) and DNA/RNA (744 and 805 cm^−1^), and inverse correlation to several AAs. CDH5 participates in VEGF‐R2 and PI3K signaling to mediate cancer progression^87^; these pathways contribute to increased membrane lipid production and the consumption of AAs to facilitate signaling. We also see procollagen C‐protease enhancer (PCOLCE) has strong inverse correlation to NAR (*r* = −0.65). While the mechanisms of PCOLCE are not as well characterized in rectal cancer, it mediates immune cell infiltration across multiple cancers.^89^, ^90^ Overall, these differentially expressed proteins and corresponding pathways and their correlation to metabolites highlight the relevance of metabolic‐proteomic joint analysis in cancer therapeutic response.

## 3 CONCLUSIONS

In summary, this study highlights the utility of RS in metabolic profiling in both PDAC and LARC patient sera to identify metabolites as potential serum biomarkers for cancer diagnosis and prediction of response, regardless of the cancer type. Our findings show RS identified key metabolites that differentiated PDAC patients from healthy patients without cancer, as well as differentiated PDAC from chronic pancreatitis, which is notoriously difficult to discriminate through current clinical analysis. We found pathways involving lipids and FAs and those associated with AAs that distinguish cancer from inflammatory phenotypes that often present similar symptoms in patients. Whereas RS was limited in differentiating LARC from healthy patients based on serum metabolites, significant changes were observed in metabolites to predict response to chemoradiation therapy and specifically differentiate poor responders pre‐ and post‐treatment. Our findings suggest that metabolic monitoring in cancer patient sera could serve as a non‐invasive assay for predicting response to therapies and complement current clinical procedures. Our correlation analysis of signaling proteins and metabolites in LARC patient sera showed joint and inverse correlations before and after chemoradiation therapy, highlighting that a collective analysis of serum proteins and metabolites is necessary to accurately predict response and/or resistance. The integration of lymphocyte DDR markers with serum signaling proteins and metabolites demonstrated that each of these markers of response is tightly regulated and intercorrelated in the TME that effectively differentiating responders from poor responders to treatment. Further, joint metabolic‐signaling pathways indicated that pre‐treatment glucose/sugars and FAs/lipids are key metabolites that contribute to cancer progression, whereas post‐treatment p53 signaling and cell cycle regulation are essential pathways that mediate response. Our analysis also indicates that both effector and memory T cells likely play a role in enabling response to chemoradiation. Furthermore, label‐free proteomics of LARC post‐treatment samples identified several key markers of cancer progression and immune infiltration, demonstrating the importance of immune‐mediated response in LARC patient treatment. Through this study, we anticipate that an integrated metabolic‐protein analysis may be leveraged in resource‐limited settings, as affordable portable Raman spectrometers are now commercially available, and label‐free Raman measurements require minimal sample preparation and a mere 5 μL of sample volume. We note that this work has some limitations, as samples for our study were received from academic biobanks that continuously bank patient biofluids for research and/or education purposes. Patients were not specifically recruited for the purposes of this work. Our aim was to demonstrate the strengths of Raman spectral metabolic profiling combined with analysis of protein signatures to enable a powerful predictive tool of treatment response that will identify early which patients are to benefit from standard of care therapies. We expect that a future focused study with a larger cohort of patient samples will improve these findings.

## 4 MATERIALS AND METHODS

### 4.1 Healthy, PDAC, and CP patient sample acquisition and ethical statement

Samples for the healthy, pancreatic ductal adenocarcinoma (PDAC), and CP groups were obtained from the Nebraska Biobank. Samples were collected under consent for research and deidentified to remove all identifiable information. As the Nebraska Biobank continuously recruits participants, the patients were not specifically recruited for this study. Blood was collected in serum separating tubes. The Nebraska biobank is an open resource for investigators and is not associated with an IRB.

### 4.2 LARC patient sample acquisition and ethical statement

The patient samples analyzed in this study (serum) were obtained from the FCCC Biosample Repository Facility (BRF) under IRB 22‐9925 and available data from https://doi.org/10.1101/2024.11.22.24317789. All patients were diagnosed with non‐metastatic, locally advanced rectal adenocarcinoma (T3/4 or node‐positive) and treated with nCRT. Radiation treatment consisted of 50–50.4Gy of external beam radiation therapy delivered in 25–28 fractions over 5–5.5 weeks. Patients received concurrent 5‐fluorouracil delivered via continuous infusion (225 mg/m^2^) or oral capecitabine (825 mg/m^2^ twice daily) on days of radiation. Surgical resection was performed ~8–10 weeks after nCRT completion. Patients were selected for analysis to provide a distribution of nCRT responses and NAR scores.

### 4.3 Patient sample preparation

Anonymized samples (0.1–1 mL patient sera) were received frozen at Iowa State University and stored at −80°C prior to aliquoting. Samples were thawed and aliquoted. Excess aliquots were flash frozen in liquid nitrogen for storage at −80°C, while samples for Raman imaging were immediately prepared. Patient sera were mixed in a 1:1 ratio with milli‐*q* water to dilute the biofluid, and 3 μL of diluted serum was pipetted onto a 2 mm diameter calcium fluoride (CaF_2_) substrate. The substrate was dried at 37°C for 25–30 min to produce a film for RS imaging.

### 4.4 Patient sample Raman spectral acquisition

RS measurements were conducted with a Renishaw inVia confocal RS microscope, using a ×50 Leica focus with a numerical aperture of 0.50. Each measurement consisted of two 10 s accumulations for a total of 20 s exposure. 10 line maps of 10 points each were collected for a total of 100 points, with a total duration of approximately 35 min per sample. Raman spectra were processed using a custom Matlab processing pipeline, consisting of cosmic ray correction, Savitzky–Golay filtering with a 5th‐order polynomial and a frame‐width of 17, and fluorescence background correction using a modified polyfit method with a 9th‐order polynomial. The processed spectra were normalized using the standard variate method (mean–variance normalization) and were then subjected to ratiometric analysis using the 1447 cm^−1^ peak as a denominator. The peaks of the normalized spectra were then quantified for further analysis.

### 4.5 Patient serum signaling proteins analysis

Serum was isolated from whole blood using standard protocols and frozen at −80°C until examination. Circulating cytokines and growth factors were evaluated using the Bio‐Plex Pro™ Human Cytokine 27‐plex Assay: #M500KCAF0Y and the Bio‐Plex Pro™ Human Cytokine 17‐plex Assay: #M5000031YV (Bio‐Rad, Hercules, CA). The assays were performed according to the manufacturer's instructions. BioPlex Manager 6.0 software was used for data analysis.

### 4.6 DDR‐related protein markers

DNA damage repair marker data from LARC patients is available from our prior work for the following protein analytes: total ATR, Chk1S345, Chk2T68, *γ*H2AXS139, p53S15, total MDM2, and total p21.^17^ Briefly, this data was generated using the MILLIPLEX MAP 7‐plex DNA Damage/Genotoxicity panel: #48‐621MAG (Millipore Sigma).^17^

### 4.7 LC–MS label‐free serum proteomics

Patient serum samples were diluted in 50 mM ammonium carbonate buffer (Fisher Scientific) pH corrected to pH 8 with 1 N HCl (Fisher Scientific) and protein concentrations were measured in technical duplicates with a BCA assay kit (Invitrogen) using a SpectraMax iD3 microplate reader at 562 nm. Using the assessed concentration, serum samples were diluted to a concentration of 1 μg/μL and submitted to the Protein Facility of the Iowa State University Office of Biotechnology. The samples were reduced with dithiothreitol (DTT), cysteine groups were modified with iodoacetamide, and were then digested overnight with trypsin and endoproteinase Lys‐C. Digestion was halted with the addition of formic acid, and the samples were dried down in a SpeedVac. The samples were then desalted using C18 columns (Nest Group BioPureSPN Mini, HUM S18V) and were redried in a SpeedVac. PRTC standard (Pierce part #88320) was spiked into the sample to serve as an internal control. The peptides were then separated by liquid chromatography using a Thermo Scientific TM Vanquish NEO coupled with an Easy‐Spray source. The separated peptides were then analyzed through MS/MS by fragmenting each peptide. The resulting intact and fragmentation pattern was compared to a theoretical fragmentation pattern to find peptides that can be used to identify the proteins. The PRTC areas were used to normalize the data between samples. To identify proteins, Proteome Discoverer 3.2 was used with the CHIMERYS algorithm.

### 4.8 Statistical analysis methods

All quantifications of error and error bars presented indicate one standard deviation. For quantification of RS peak analysis, unpaired, two‐sided, homoscedastic student's *t*‐tests were used to generate *p*‐values, where not significant (ns) indicates *p*‐value ≥0.05, * indicates *p*‐value <0.05, ** indicates *p*‐value <0.01, and *** indicates *p*‐value <0.001, and **** indicates *p*‐value <0.0001. Pairwise Pearson's correlations were calculated using the “corrcoef” function from the numpy library (Python 3.12.4). PCA and t‐stochastic neighbor embedding (tSNE) were performed using the Matlab Machine Learning and Statistics Toolbox pca() and tsne() functions, respectively. SVM analysis was performed using the Python 3.12.4 scikit‐learn svc() function with a linear kernel, auto‐sale, and a c‐value hyperparameter optimized between 1 and 100 using leave‐one‐out cross validation and test accuracy as the ranking metric.

### 4.9 Metaboanalyst 6.0

MetaboAnalyst 6.0, a web platform for the analysis of metabolic and related ‘omics data, was used to perform quantitative enrichment analysis and joint pathway analysis. Quantitative enrichment analysis and joint pathway analysis were performed using the KEGG pathway database. Here, the intensity of the Raman peaks for the assigned metabolite was used as a “proxy” for metabolite concentrations to uncover the metabolic pathways. This proxy was used as the Raman peak intensity is linearly correlated to the concentration of measured analytes in sera based on our previous validation work. Joint pathway analysis used closeness centrality and pooled queries for the identification of both impacted genetic and metabolic pathways.

### 4.10 WebGestalt 2024 for Gene set enrichment analysis (GSEA)

WebGestalt 2024, a web platform for the analysis of genomic, proteomic, and related ‘omics data, was used to perform ontology and GSEA for pooled protein marker and proteomics data. The GSEA rank metric was calculated with the formula below:

$$\text{GSEA}\;\mathrm{rank}\;\text{metric}=-\log_{10}p\times \text{SIGN}\left(\log_{2}\mathrm{FC}\right)$$

where *p* is the *p*‐value calculated by an unpaired, two‐sided, homoscedastic student's *t*‐test, FC is the fold change between the complete and poor responder groups, and the SIGN function returns the sign of the base‐2 log of the fold change. Proteins were mapped using NCBI Entrez Gene Ids.

## AUTHOR CONTRIBUTIONS

RB conceived the project idea, directed the study design, and edited the manuscript. GC trained EQ, NL, and AA, performed Raman measurements, data analysis, data interpretation, and wrote the manuscript. EVD and PC performed Luminex‐based ELISA assays for inflammatory and DDR‐related markers, data analysis, and assisted with data management. EQ aided in data analysis and assisted in Raman measurements. NL and AA assisted in Raman measurements. SB provided insight on PDAC and provided anonymized biobank samples. SA provided insight and data interpretation on LARC, provided study samples and data from LARC patients, and edited the manuscript.

## CONFLICT OF INTEREST STATEMENT

Author S.A. has the following disclosures: S.A. performs collaborative research (with no funding) with Caris Life Sciences. S.A.'s spouse is employed by Akoya Biosciences and has stocks in Akoya Biosciences, and Senzo Health. S.A. has several patents related to cancer diagnostics/treatment. These disclosures do not conflict with the present study. The other authors declare no conflict of interest.

## Supporting information


**Data S1:** Supplementary Information.

## Data Availability

The data that support the findings of this study are available from the corresponding author upon reasonable request.
