# Supplementary material for: Metabolic and proteomic signatures differentiate inflammatory phenotypes from cancer and predict treatment response in patient sera
Source: Bioeng Transl Med. 2025 May 15;10(5):e70029. doi: 10.1002/btm2.70029 (PMC12478453; doi:10.1002/btm2.70029)
Supplement: Supplementary file 1 — Data S1: Supplementary Information. [file BTM2-10-e70029-s001.docx]

**Supporting Information**

**Metabolic and Proteomic Signatures Differentiate Inflammatory Phenotypes from Cancer and Predict Treatment Response in Patient Sera**

Gabriel Cutshaw^a,b^, Elena V. Demidova^c^, Philip Czyzewicz^c^, Elizabeth Quam^a,b^, Nicole Lorang^a,b^, AL warith AL siyabi^a,b^, Surinder Batra^d^, Sanjeevani Arora^c,e,f^*, Rizia Bardhan^a,b,^*

a Department of Chemical and Biological Engineering, Iowa State University, Ames, IA 50011, USA

b Nanovaccine Institute, Iowa State University, Ames, IA 50012, USA

c Cancer Prevention and Control Program, Fox Chase Cancer Center, PA 18925, USA

d Department of Biochemistry and Molecular Biology, University of Nebraska Medical Center, NE 68198, USA

e Cancer Epigenetics Institute, Fox Chase Cancer Center, PA 18925, USA

f Department of Radiation Oncology, Fox Chase Cancer Center, PA 18925, USA

*Corresponding authors: [rbardhan@iastate.edu](mailto:rbardhan@iastate.edu); sanjeevani.arora@fccc.edu

**Table of Contents**

**Figure S1**. Raman average peak values and PCA plot for healthy, PDAC, and LARC patients…...3

**Figure S2**. Raman average peak values for healthy, PDAC, and chronic pancreatitis patients……4

**Figure S3**. PCA-tSNE and classical univariate AUC-ROC analysis for differentiating PDAC and chronic pancreatitis……………………………………………..………………………….…...…5

**Figure S4.** Quantitative enrichment analysis comparison for pancreatic cancer versus control and LARC versus control patients...........................................................................................................5

**Table S1.** Metabolites obtained from Raman spectroscopy contribute to various KEGG pathway analysis. …..……………………………………………………………………………………….6

**Figure S5.** Difference spectra and Pearson’s correlation for post-treatment LARC samples…….7

**Figure S6.** Differentiation of poor and complete responder LARC patients using RS data..............8

**Figure S7.** Quantitative Pearson correlation heatmap for LARC pre-treatment patients using RS, and cytokine/chemokine data………………….………………………………………………......9

**Figure S8.** Quantitative Pearson correlation heatmap for LARC post-treatment patients using RS, and cytokine/chemokine data…………………………………………………….........................10

**Figure S9.** Differentiation of poor and complete responder LARC patients using RS and cytokine data……………………………………………………………………………………………….11

**Figure S10.** Quantitative Pearson correlation heatmap for LARC pre-treatment patients using RS, cytokine/chemokine, and gene marker data……………………………………………………....12

**Figure S11.** Quantitative Pearson correlation heatmap for LARC post-treatment patients using RS, cytokine/chemokine, and gene marker data……………………………………………………....13

**Figure S12.** Differentiation of poor and complete responder LARC patients using RS data, cytokine data and gene marker data................................................................................................14

**Table S2.** Protein and metabolic contributions to various KEGG joint-pathway analysis. For pre-treatment LARC patients…………………………………………………………………………14

**Table S3.** Protein and metabolic contributions to various KEGG joint-pathway analysis. For post-treatment LARC patients …..…………………………………………………………………….15

**Table S4.** Differentiation accuracy of LARC patient response across dataset combinations assessed with PCA-SVM and leave-one-out cross validation.........................................................15

**Figure S13.** Ontology of proteins found in post-treatment LARC patient serum through targeted label-free proteomics……………………………………………………………………………..16

**Table S5**. Protein contribution to Gene Set Analysis of post-treatment LARC patient serum with KEGG pathways. …………………………………………………………………………...……17

**Table S6.** Protein expression contribution to Gene Set Analysis of post-treatment LARC patient serum with Reactome pathways. …………………………………………………………………18

**Figure S14**. Quantitative Pearson correlation heatmap for LARC post-treatment patients using RS, cytokine/chemokine, and differentially expressed label-free proteomics data. …………………19


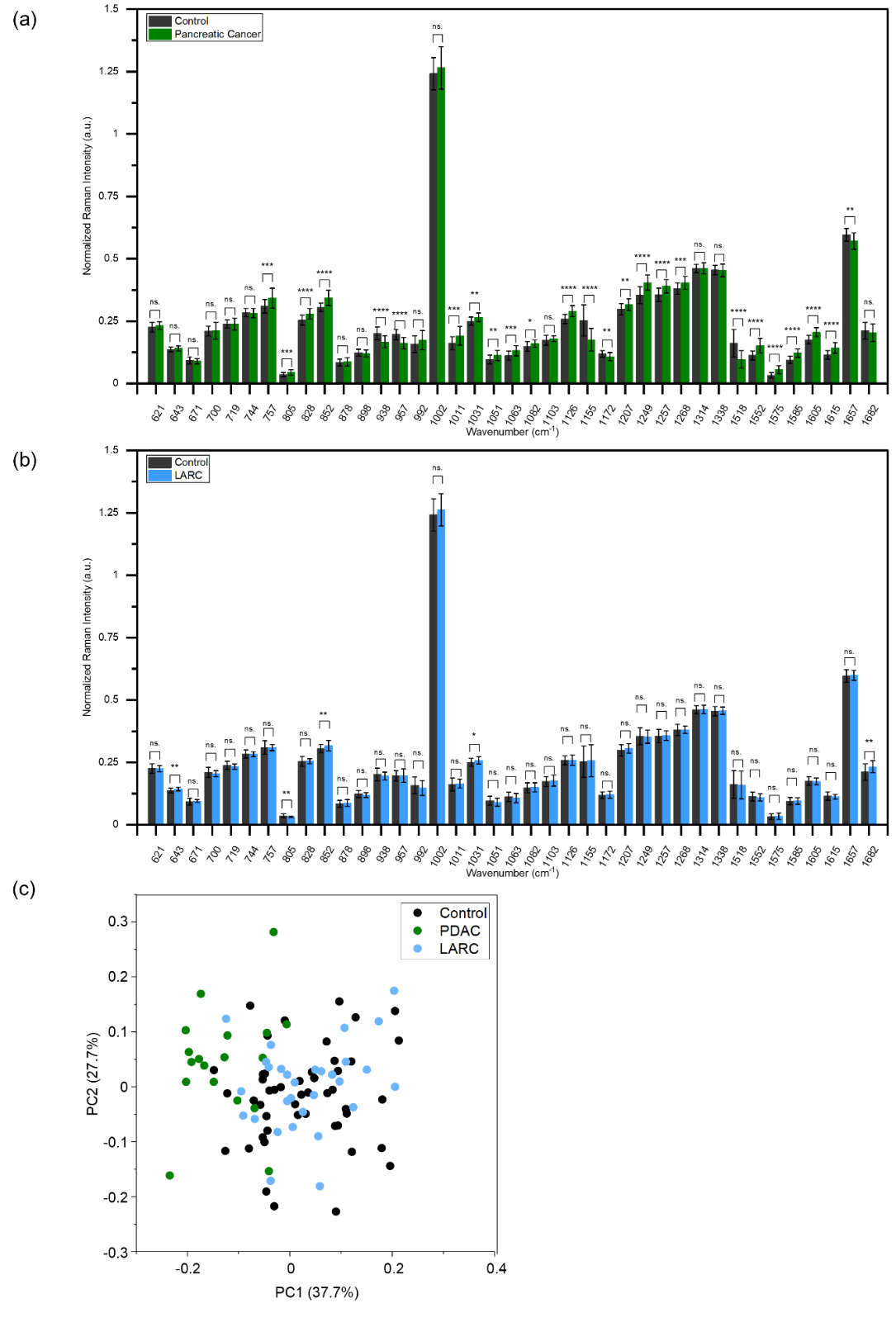


**Figure S1.** (a - b) Average RS peak value comparisons for (a) n = 18 pancreatic ductal adenocarcinoma (PDAC) patient samples and n = 48 healthy patient samples, and (b) n = 30 pre-treatment locally advanced rectal cancer (LARC) and n = 48 healthy patient samples. Significance determined with a homoscedastic two-tailed t-test (p ≥ 0.05: not significant (ns.), p < 0.05: *, p < 0.01: **, p < 0.001: ***, p < 0.0001: ****). (c) PCA plot for PC1 and PC2 for n = 48 healthy patient samples, n = 18 PDAC patient samples, and n = 30 LARC patients.


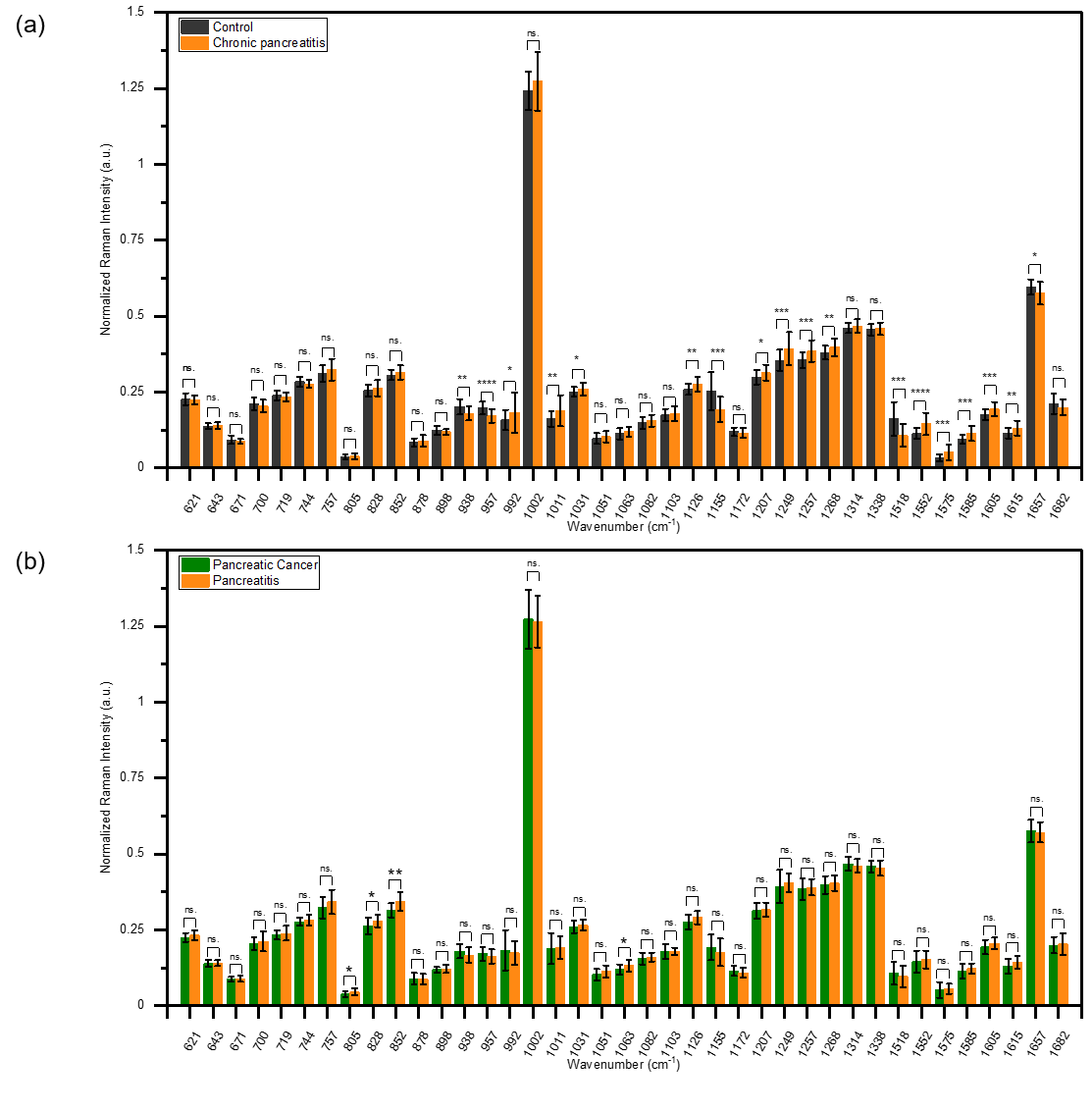


**Figure S2.** (a - b) Average RS peak value comparisons for (a) n = 20 chronic pancreatitis (CP) and n = 48 healthy patient samples, and (b) n = 18 PDAC and n = 20 CP patient samples. Significance determined with a homoscedastic two-tailed t-test (p ≥ 0.05: not significant (ns.), p < 0.05: *, p < 0.01: **, p < 0.001: ***, p < 0.0001: ****).


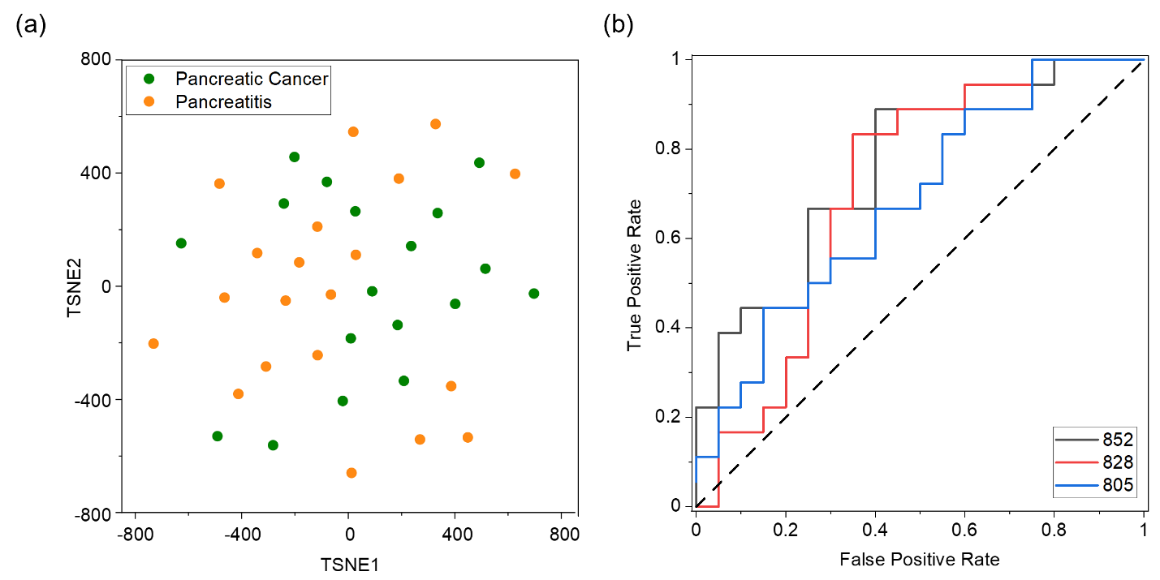


**Figure S3.** (a) PCA-tSNE plot for TSNE1 and TSNE2 for n = 18 pancreatic ductal adenocarcinoma and n = 20 chronic pancreatitis patients. (b) Classical AUC-ROC analysis for RS metabolite peaks for binary classification of pancreatic cancer versus pancreatitis patients. The peaks include the 852 cm^-1^ sugars peak (AUC = 0.76, 95% CI 0.61 – 0.91), the 828 cm^-1^ tyrosine peak (AUC = 0.72, 95% CI 0.54 – 0.88) and the 805 cm^-1^ DNA/RNA peak (AUC = 0.69, 95% CI 0.51 – 0.82).


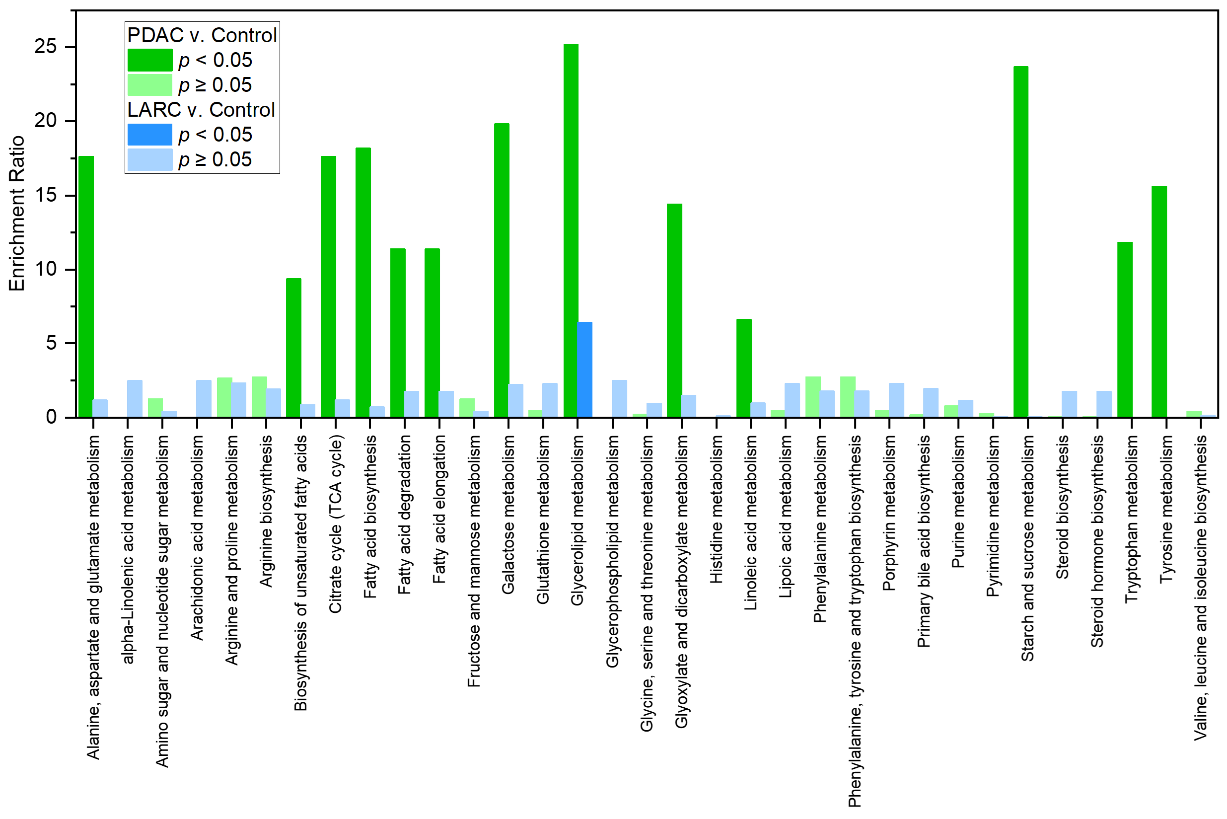


**Figure S4.** Quantitative metabolic pathway enrichment analysis for (n = 18) pancreatic cancer patients and (n = 30) pre-treatment locally advanced rectal cancer patients versus n = 48 healthy patients. Tentative RS peak assignments used as proxies for metabolite with KEGG pathway database. P-values reported by Metaboanalyst 6.0.

**Table S1.** Metabolites obtained from Raman data contribute to quantitative enrichment analysis with KEGG pathways. The total compounds and hits were obtained from Metaboanalyst 6.0.

| **Pathway** | **Total Compounds** | **Hits** | **Raman Metabolites** |
| --- | --- | --- | --- |
| Alanine, aspartate, and glutamate metabolism | 28 | 2 | Citric acid, glutamic acid |
| alpha-Linoleic acid metabolism | 13 | 1 | Phosphatidylcholine |
| Amino sugar and nucleotide sugar metabolism | 42 | 1 | D-mannose |
| Arachidonic acid metabolism | 44 | 1 | Phosphatidylcholine |
| Arginine and proline metabolism | 36 | 3 | Glutamic acid, L-arginine, proline |
| Arginine biosynthesis | 14 | 2 | Glutamic acid, L-arginine |
| Biosynthesis of unsaturated fatty acids | 36 | 3 | Arachidic acid, palmitic acid, linoleic acid |
| Citrate cycle (TCA cycle) | 20 | 1 | Citric acid |
| Fatty acid biosynthesis | 47 | 2 | Palmitic acid, myristic acid |
| Fatty acid degradation | 39 | 1 | Palmitic acid |
| Fatty acid elongation | 38 | 1 | Palmitic acid |
| Fructose and mannose metabolism | 20 | 1 | D-mannose |
| Galactose metabolism | 27 | 3 | D-glucose, d-mannose, glycerol |
| Glutathione metabolism | 28 | 2 | Glutamic acid, glycine |
| Glycerolipid metabolism | 16 | 1 | Glycerol |
| Glycerophospholipid metabolism | 36 | 1 | Phosphatidylcholine |
| Glycine, serine and threonine metabolism | 33 | 2 | Glycine, L-threonine |
| Glyoxylate and dicarboxylate metabolism | 31 | 3 | Citric acid, glutamic acid, glycine |
| Histidine metabolism | 16 | 2 | Glutamic acid, histidine |
| Linoleic acid metabolism | 5 | 2 | Linoleic acid, phosphatidylcholine |
| Lipoic acid metabolism | 28 | 1 | Glycine |
| Phenylalanine metabolism | 8 | 2 | Phenylalanine, L-tyrosine |
| Phenylalanine, tyrosine and tryptophan metabolism | 4 | 2 | Phenylalanine, L-tyrosine |
| Porphyrin metabolism | 31 | 2 | Glutamic acid, glycine |
| Primary bile acid biosynthesis | 46 | 2 | Cholesterol, glycine |
| Purine metabolism | 70 | 1 | Guanine |
| Pyrimidine metabolism | 39 | 1 | Thymine |
| Starch and sucrose metabolism | 18 | 1 | D-glucose |
| Steroid hormone biosynthesis | 87 | 1 | Cholesterol |
| Tryptophan metabolism | 41 | 1 | L-tryptophan |
| Tyrosine metabolism | 42 | 1 | L-tyrosine |


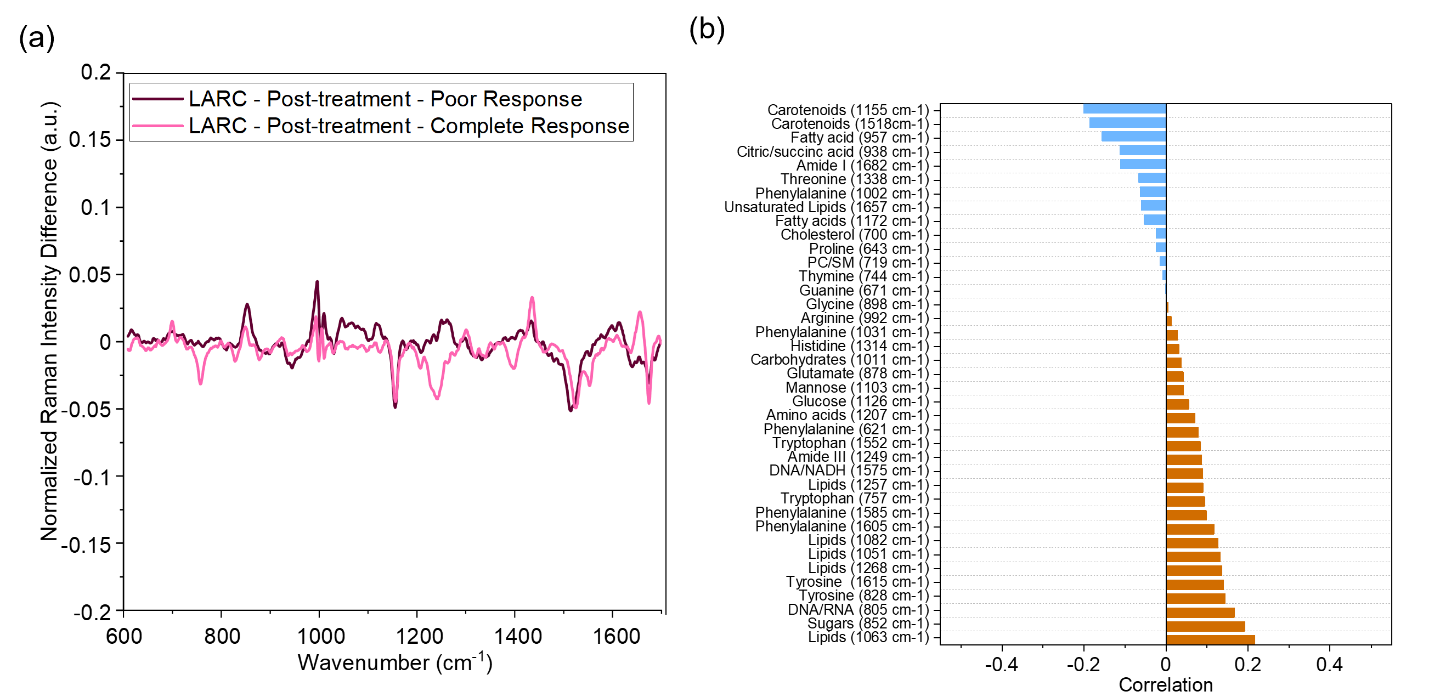


**Figure S5.** (a) Serum RS difference spectra for post-treatment locally advanced rectal cancer (LARC) complete responder samples and post-treatment LARC poor responder samples versus healthy patient samples. Difference spectra were obtained by subtracting the representative healthy patient spectrum from the representative LARC spectra. (b) Pearson’s correlation of RS peaks against NAR score for n = 34 complete responder, n = 7 partial responder and n = 44 poor responder LARC patients.


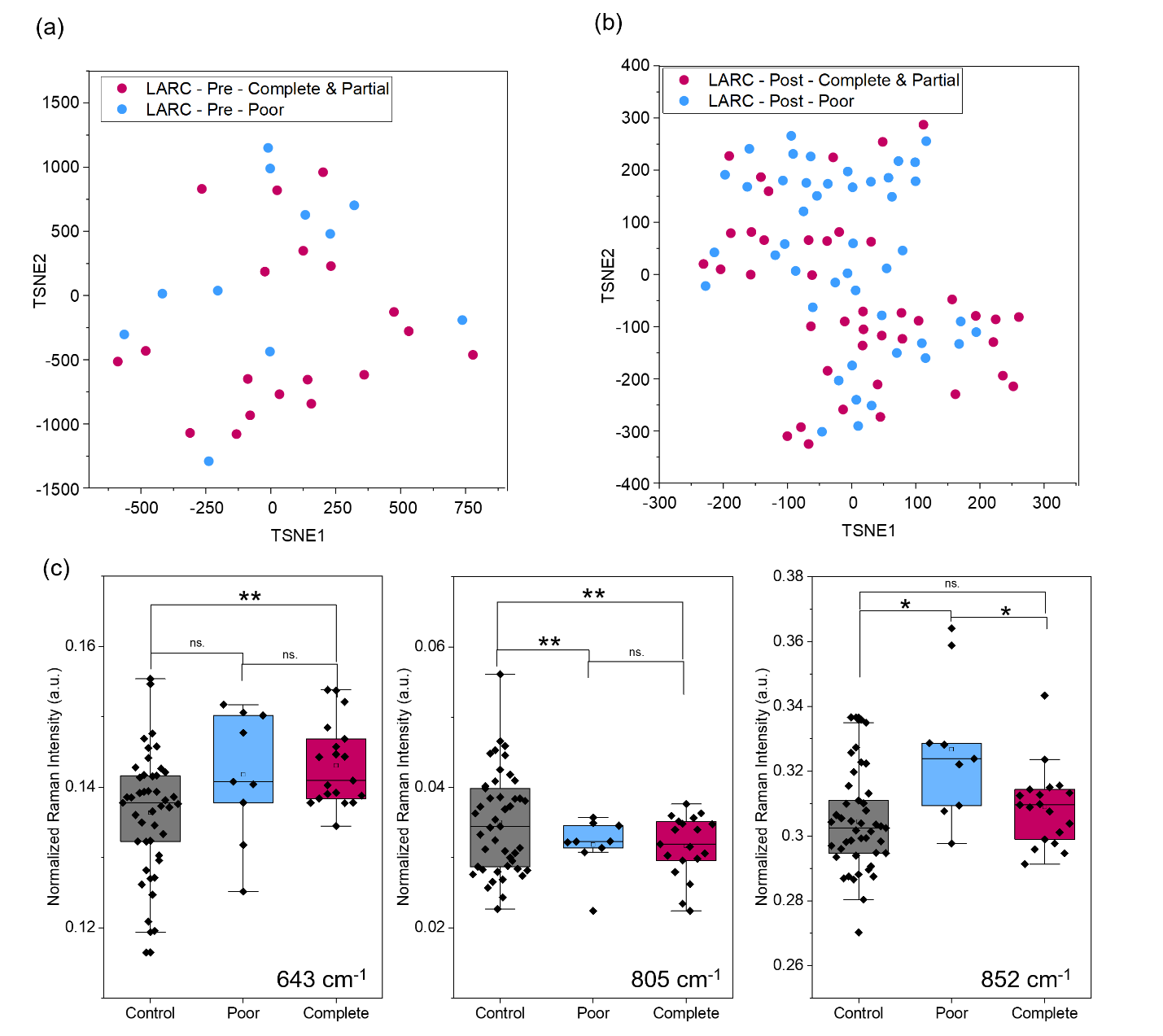


**Figure S6.** (a - b) PCA-tSNE differentiation for RS peak data for (a) n = 11 pre-treatment poor responder and n = 19 pre-treatment complete & partial responder and (b) n = 44 post-treatment poor responder and n = 41 post-treatment complete & partial responder. (c) Selected RS metabolite peak values for n = 19 complete or partial responder and n = 11 poor responder LARC pre-treatment patient samples for key metabolic peaks. Significance determined with a homoscedastic two-tailed t-test (p > 0.05: not significant (ns.), p < 0.05: *, p < 0.01: **, p < 0.001: ***, p < 0.0001: ****).


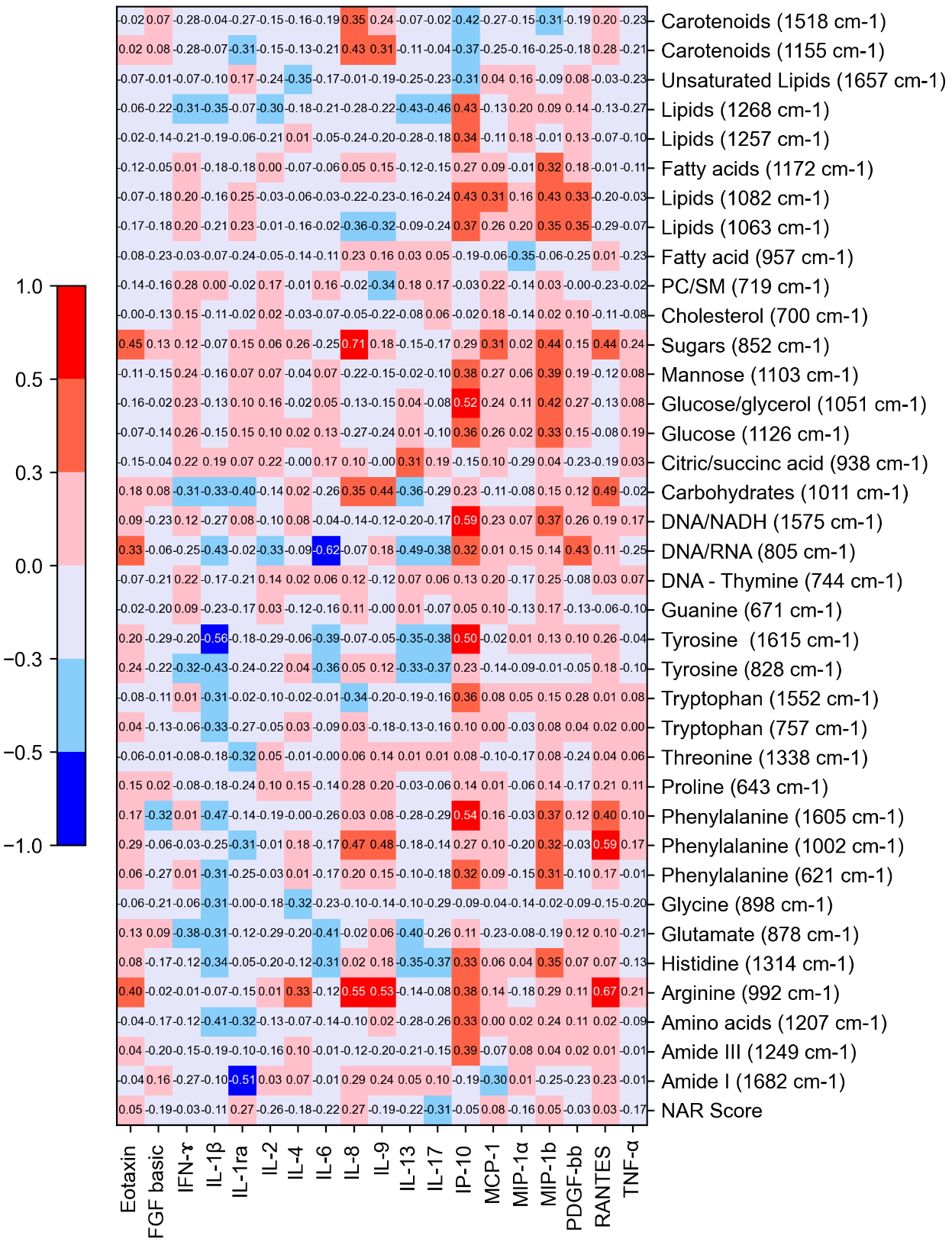


**Figure S7.** Pre-treatment Pearson correlation heatmap of RS peaks to serum cytokines and chemokines. Correlations were conducted with for n = 8 complete responder (NAR < 8), n = 9 partial responder (8 < NAR < 14) and n = 7 poor responder (NAR > 14) LARC patients. For post-treatment, correlations were conducted with n = 17 complete responder, n = 7 partial responder and n = 17 poor responder LARC patients.

**
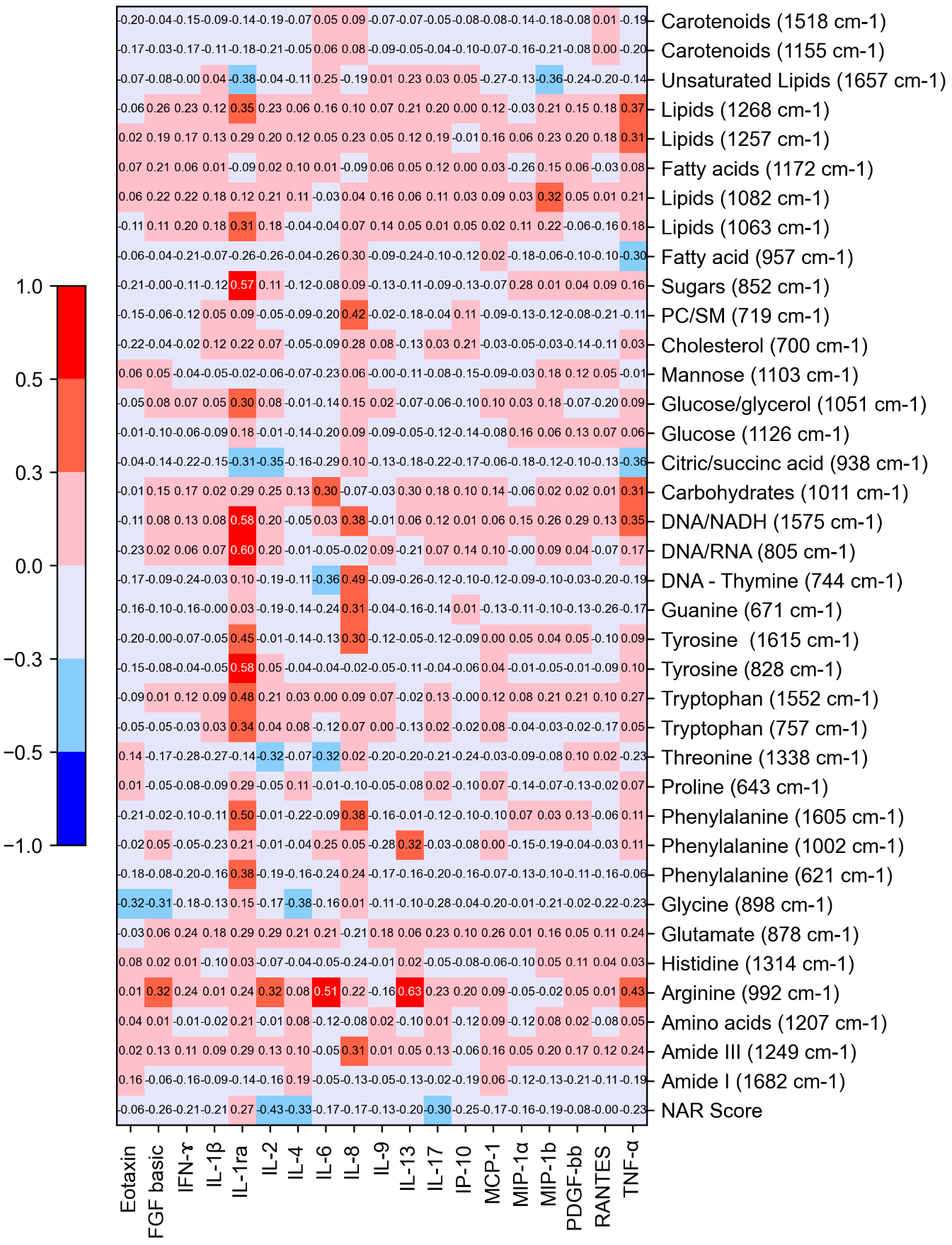
**

**Figure S8.** Post-treatment Pearson correlation heatmap of RS peaks to serum cytokines and chemokines. Correlations were conducted with n = 17 complete responder, n = 7 partial responder and n = 17 poor responder LARC patients.


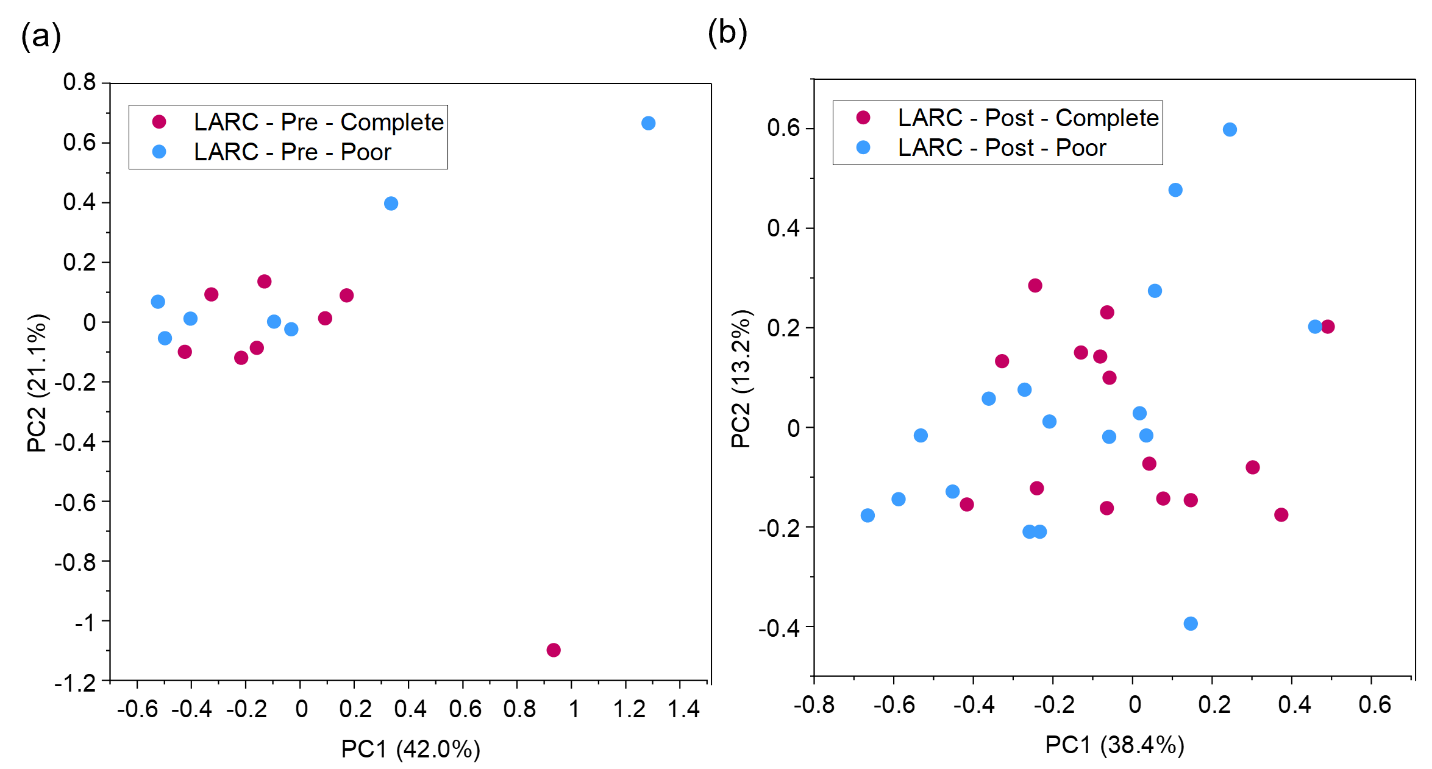


**Figure S9.** (a - b) PCA differentiation for RS peak + cytokine/chemokine data for (a) n = 7 pre-treatment poor responder and n = 8 pre-treatment complete responder and (b) n = 17 post-treatment poor responder and n = 17 post-treatment complete responder.


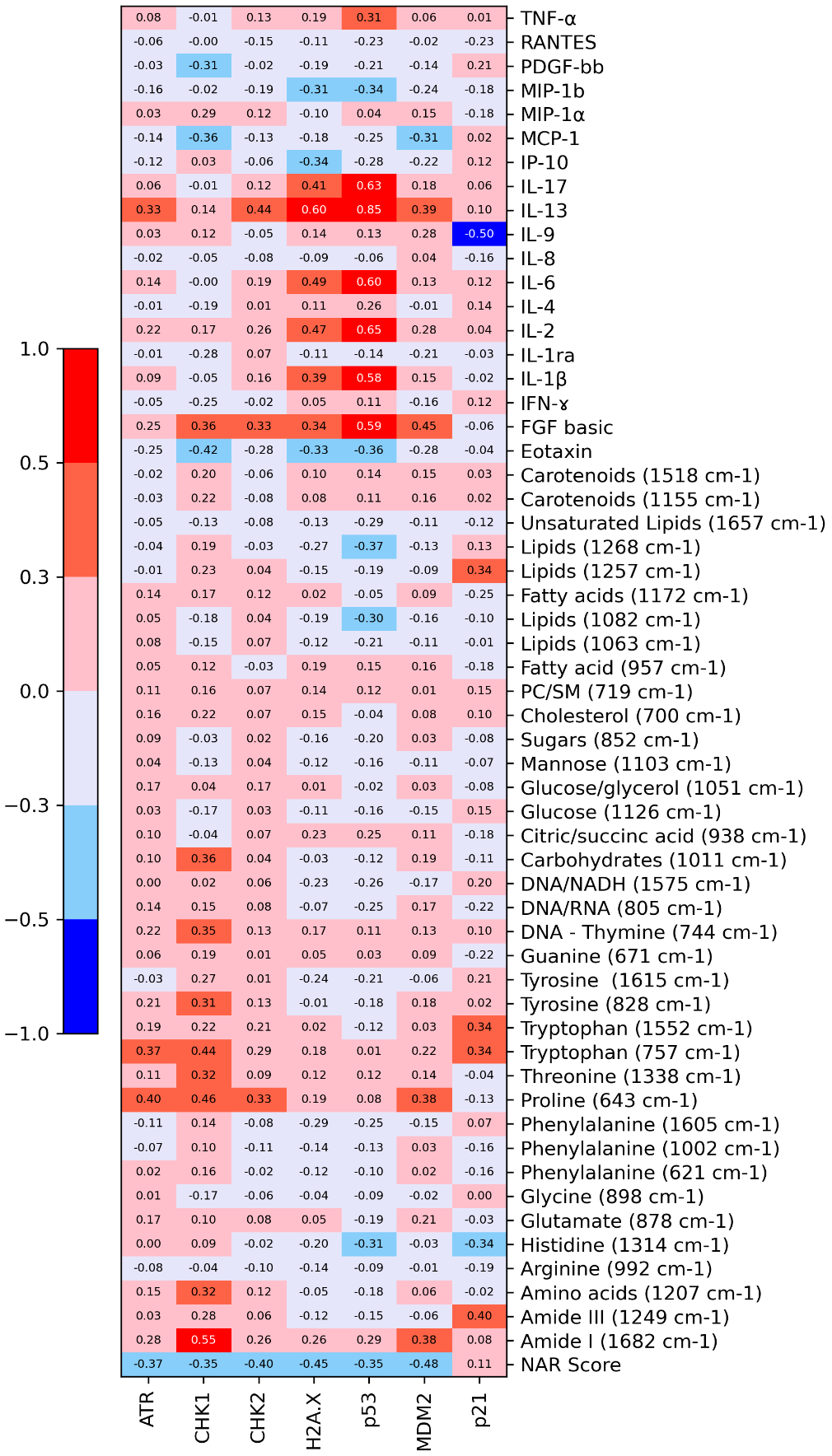


**Figure S10.** Pre-treatment Pearson correlation heatmap of lymphocyte gene markers to serum RS metabolites, serum cytokines and serum chemokines. Correlations were conducted with for n = 8 complete responder (NAR < 8), n = 8 partial responder (8 < NAR < 14) and n = 7 poor responder (NAR > 14) LARC patients.


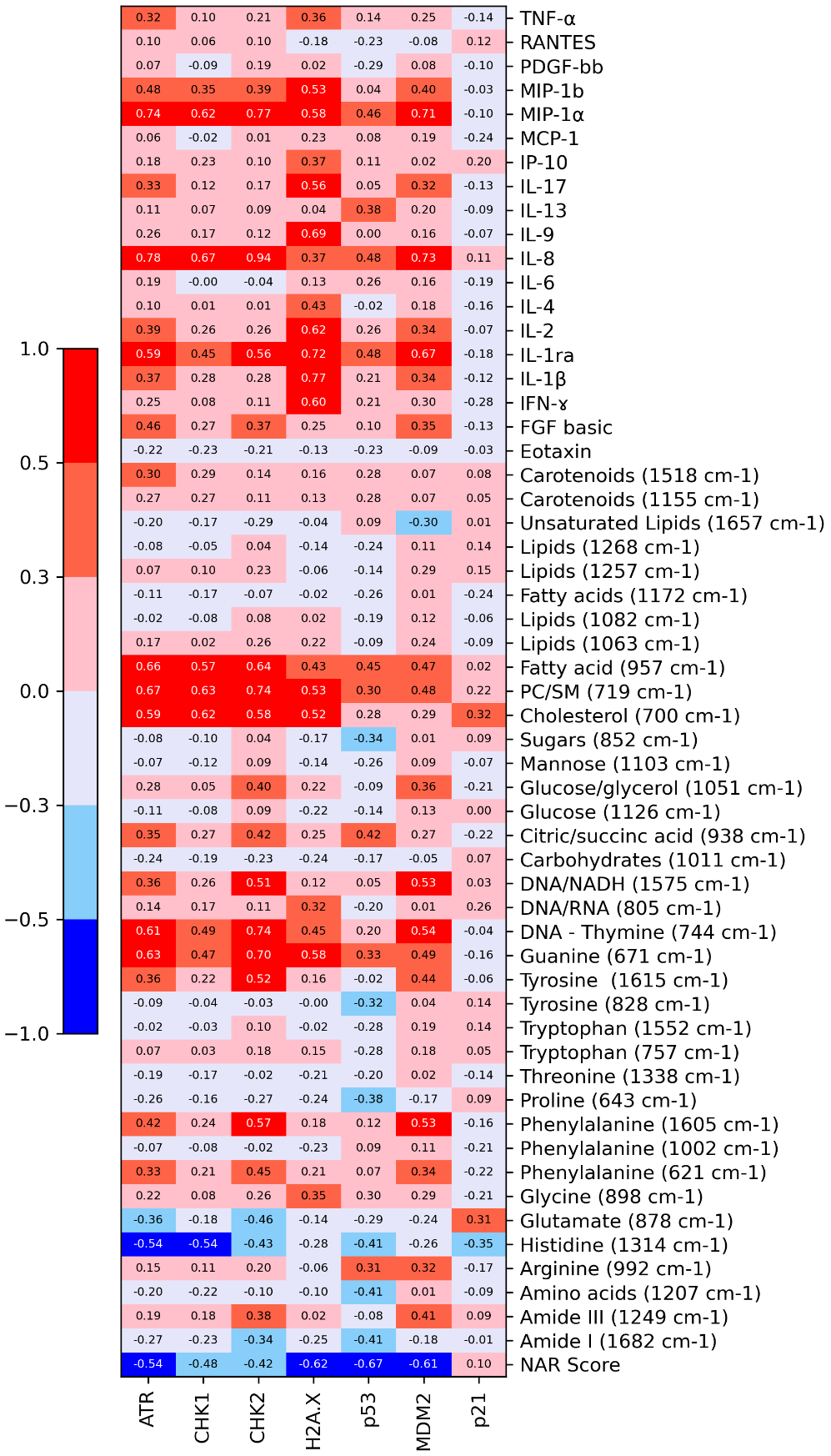


**Figure S11.** Post-treatment Pearson correlation heatmap of lymphocyte gene markers to serum RS metabolites, serum cytokines and serum chemokines. Correlations were conducted with n = 9 complete responder and n = 9 poor responder LARC patients.


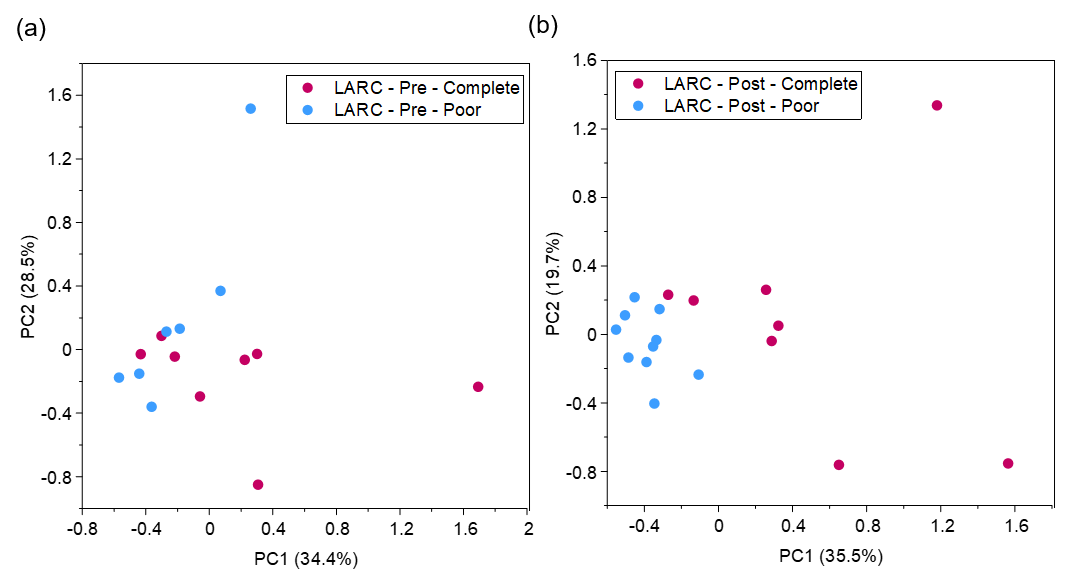


**Figure S12.** (a - b) PCA differentiation for RS peak + cytokine/chemokine + gene marker data for (a) n = 7 pre-treatment poor responder and n = 8 pre-treatment complete responder and (b) n = 8 post-treatment poor responder and n = 10 post-treatment complete responder.

**Table S2.** Significant differentiating pathways and their matched features for LARC pre-treatment joint-pathway analysis obtained from Raman spectroscopy contribute to various KEGG pathway analysis. The total match status was obtained from Metaboanalyst 6.0.

| **Pathway** | **Total Nodes** | **Hits** | **Matched Features** |
| --- | --- | --- | --- |
| Cell cycle | 124 | 3 | CHK1, CHK2, MDM2 |
| Cellular senescence | 165 | 3 | CHK1, CHK2, MDM2 |
| Endocrine resistance | 98 | 1 | MDM2 |
| Galactose metabolism | 77 | 1 | Glycerol |
| p53 signaling pathway | 72 | 3 | CHK1, CHK2, MDM2 |
| Platinum drug resistance | 76 | 1 | MDM2 |

**Table S3.** Significant differentiating pathways and their matched features for LARC post-treatment joint-pathway analysis obtained from Raman spectroscopy contribute to various KEGG pathway analysis. The total match status was obtained from Metaboanalyst 6.0.

| **Pathway** | **Total Nodes** | **Hits** | **Matched Features** |
| --- | --- | --- | --- |
| Cell cycle | 124 | 5 | ATR, CHK1, CHK2, MDM2, p21 |
| Cellular senescence | 165 | 5 | ATR, CHK1, CHK2, MDM2, p21 |
| C-type lectin receptor signaling pathway | 104 | 2 | IL-2, MDM2 |
| Cytokine-cytokine receptor interaction | 294 | 4 | IL-1ra, IL-2, IFN-γ, MIP-1α |
| FoxO signaling pathway | 131 | 2 | MDM2, p21 |
| Endocrine resistance | 98 | 2 | MDM2, p21 |
| HIF-1 signaling pathway | 109 | 2 | IFN-γ, p21 |
| JAK-STAT signaling pathway | 162 | 3 | IL-2, IFN-γ, p21 |
| Necroptosis | 162 | 2 | H2A.X, IFN-γ |
| p53 signaling pathway | 72 | 5 | ATR, CHK1, CHK2, MDM2, p21 |
| PI3K-Akt | 354 | 3 | IL-2, MDM2, p21 |
| Platinum drug resistance | 73 | 2 | MDM2, p21 |
| T cell receptor signaling pathway | 104 | 2 | IL-2, IFN-γ |
| Th1 and Th2 cell differentiation | 92 | 2 | IL-2, IFN-γ |
| Th17 cell differentiation | 107 | 2 | IL-2, IFN-γ |
| Transcriptional misregulation in cancer | 186 | 2 | MDM2, p21 |

**Table S4.** PCA-SVM differentiation accuracy using leave-one-out cross validation (LOOCV) for collected datasets. Proteomics feature set shows significant overfitting errors due to disproportionate feature-to-sample ratio.

| **Dataset** | **Pre-treatment CV accuracy** | | **Post-treatment CV accuracy** | |
| --- | --- | --- | --- | --- |
|  | **Test** | **Train** | **Test** | **Train** |
| RS metabolites | 57.9% | 57.9% | 61.1% | 62.5% |
| Signaling proteins | 66.7% | 71.9% | 58.8% | 63.4% |
| Label-free proteomics |  |  | 27.8% | 44.8% |
| RS metabolites + signaling proteins | 60.0% | 71.0% | 58.8% | 63.3% |
| RS metabolites + label free proteomics |  |  | 27.8% | 60.1% |
| RS metabolites + signaling proteins + DNA damage markers | 73.3% | 75.2% | 94.4% | 93.8% |
| RS + signaling proteins + DNA damage markers + label-free proteomics |  |  | 33.3% | 59.8% |


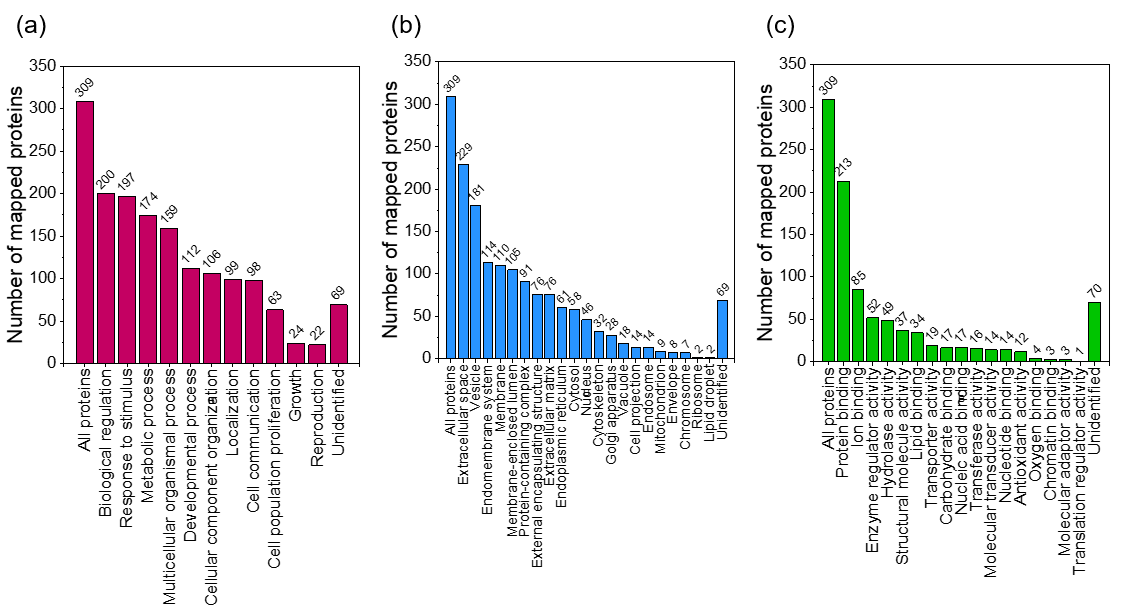


**Figure S13.** (a – c) Ontology of collated and gene id mapped serum proteins, signaling proteins, and DNA damage markers for post-treatment LARC patients. Ontology reported via WebGestalt 2024 using the GO Slim subset. Proteins are categorized here based on (a) biological process, (b) cellular components and (c) molecular function.

**Table S5.** Differentiating pathways and their matched features for LARC post-treatment Gene Set Analysis (GSEA) with **KEGG pathways**. Features reported using official HGNC gene symbols. The normalized enrichment score, size and leading edges were obtained from WebGetsalt 2024. Features are in bold text and GSEA rank metric for each feature is in parentheses.

| **Pathway** | **Size** | **Leading edge number** | **Leading edge features** |
| --- | --- | --- | --- |
| p53 signaling pathway | 8 | 5 | **TP53** (4.15), **MDM2** (3.25), **ATR** (2.64), **CHEK1** (1.88), **CHEK2** (1.56) |
| Cellular senescence | 9 | 6 | **TP53** (4.15), **MDM2** (3.25), **ATR** (2.64), **CHEK1** (1.88), **CHEK2** (1.56), **IL6** (1.08) |
| Cell cycle | 6 | 5 | **TP53** (4.15), **MDM2** (3.25), **ATR** (2.64), **CHEK1** (1.88), **CHEK2** (1.56) |
| Cytokine-cytokine receptor interaction | 19 | 14 | **IL1RN** (1.86), **CCL3** (1.46), **IFNG** (1.44), **IL2** (1.36), **IL6** (1.08), **IL1B** (1.03), **IL17A** (0.81), **IL13** (0.76), **CXCL10** (0.68), **TNF** (0.67) |
| Inflammatory bowel disease | 8 | 7 | **IFNG** (1.44), **IL2** (1.36), **IL6** (1.08), **IL1B** (1.03), **IL17A** (0.81), **IL13** (0.76), **TNF** (0.67) |
| Fluid shear stress and atherosclerosis | 10 | 6 | **TP53** (4.15), **CDH5** (1.92), **IFNG** (1.44), **IL1B** (1.03), **TNF** (0.67), **CCL2** (0.50) |
| Phagosome | 10 | 7 | **FCGR3A** (-.75), **COMP** (-0.54), **CD14** (-0.43), **THBS1** (-0.34), **C1R** (-0.21), **TUBA4A** (-0.20), **ACTB** (-0.16) |
| ECM-receptor interaction | 9 | 7 | **CD44** (-0.89), **COMP** (-0.54), **THBS1** (-0.34), **FN1** (-0.33), **GP1BA** (-0.29), **GP5** (-0.26), **VWF** (-0.20) |
| Platelet activation | 8 | 8 | **FGB** (-0.88), **FGG** (-0.67), **F2** (-0.57), **FGA** (-0.35), **GP1BA** (-0.29), **GP5** (-0.26), **VWF** (-0.20), **ACTB** (-0.16) |
| Carbon metabolism | 6 | 3 | **PGK1** (-0.58), **GADPH** (-0.45), **PGAM2** (-0.42) |
| Biosynthesis of amino acids | 6 | 3 | **PGK1** (-0.58), **GADPH** (-0.45), **PGAM2** (-0.42) |
| Complement and coagulation cascades | 52 | 14 | **F13B** (-1.85), **CFHR2** (-1.34), **CFH** (-0.97), **FGB** (-0.88), **MASP1** (-0.85), **F13A1** (-0.83), **CPB2** (-0.76), **C2** (-0.70), **FGG** (-0.67), C6 (-0.64) |
| Glycolysis / Gluconeogenesis | 7 | 4 | **MINPP1** (-1.40), **PGK1** (-0.58), **GADPH** (-0.45), **PGAM2** (-0.42) |
| Thyroid hormone synthesis | 5 | 2 | **GPX3** (-0.69), **SERPINA7** (-0.68) |
| Metabolic pathways | 16 | 9 | **MINPP1** (-1.40), **PTGDS** (-0.81), **GPX3** (-0.69), **CKM** (-0.64), **PGK1** (-0.58), **GPLD1** (-0.5), **BTD** (-0.47), **GADPH** (-0.45), **PGAM2** (-0.42) |

**Table S6.** Differentiating pathways and their matched features for LARC post-treatment Gene Set Analysis (GSEA) with **Reactome pathways**. Features reported using official HGNC gene symbols. The normalized enrichment score, size and leading edges were obtained from WebGetsalt 2024. Features are in bold text and GSEA rank metric for each feature is in parentheses.

| **Pathway** | **Size** | **Leading edge number** | **Leading edge features** |
| --- | --- | --- | --- |
| Generic Transcription Pathway | 15 | 9 | **TP53** (4.15), **H2AX** (3.69), **MDM2** (3.25), **ATR** (2.64), **CHEK1** (1.88), **CHEK2** (1.56), **IFNG** (1.44), **IL2** (1.36), **IL6** (1.08) |
| RNA Polymerase II Transcription | 15 | 9 | **TP53** (4.15), **H2AX** (3.69), **MDM2** (3.25), **ATR** (2.64), **CHEK1** (1.88), **CHEK2** (1.56), **IFNG** (1.44), **IL2** (1.36), **IL6** (1.08) |
| Gene expression (Transcription) | 16 | 9 | **TP53** (4.15), **H2AX** (3.69), **MDM2** (3.25), **ATR** (2.64), **CHEK1** (1.88), **CHEK2** (1.56), **IFNG** (1.44), **IL2** (1.36), **IL6** (1.08) |
| Cellular responses to stress | 18 | 7 | **TP53** (4.15), **H2AX** (3.69), **MDM2** (3.25), **ATR** (2.64), **HBB** (1.17), **IL6** (1.08), **APOB** (0.98) |
| Cellular responses to stimuli | 18 | 7 | **TP53** (4.15), **H2AX** (3.69), **MDM2** (3.25), **ATR** (2.64), **HBB** (1.17), **IL6** (1.08), **APOB** (0.98) |
| Signaling by Interleukins | 28 | 16 | **TP53** (4.15), **IL1RN** (1.86), **CCL3** (1.46), **IFNG** (1.44), **IL2** (1.36), **IL6** (1.08), **IL1B** (1.03), **FGF2** (0.86), **IL17A** (0.81), **IL13** (0.76) |
| Cell Cycle | 8 | 6 | **TP53** (4.15), **H2AX** (3.69), **MDM2** (3.25), **ATR** (2.64), **CHEK1** (1.88), **CHEK2** (1.56) |
| Cell Cycle Checkpoints | 7 | 6 | **TP53** (4.15), **H2AX** (3.69), **MDM2** (3.25), **ATR** (2.64), **CHEK1** (1.88), **CHEK2** (1.56) |
| DNA Repair | 6 | 5 | **TP53** (4.15), **H2AX** (3.69), **ATR** (2.64), **CHEK1** (1.88), **CHEK2** (1.56) |
| Interleukin-10 signaling | 9 | 9 | **IL1RN** (1.86), **CCL3** (1.46), **IL6** (1.08), **IL1B** (1.03), **CXCL10** (0.68), **TNF** (0.67), **CXCL8** (0.61), **CCL2** (0.5), **CCL4** (0.46) |
| Metabolism | 35 | 11 | **MINPP1** (-1.40), **CD44** (-0.89), **PTGD2** (-0.81), **PRSS1** (-0.74), **CKM** (-0.64), **APOE** (-0.59), **PGK1** (-0.58), **BTD** (-0.47), **GAPDH** (-0.45), **PGAM2** (-0.42), **APOM** (-0.39) |
| Neutrophil degranulation | 33 | 6 | **PPBP** (-1.89), **LYZ** (-1.46), **CST3** (-1.27), **S100A9** (-0.96), **CD44** (-0.89), **SELL** (-0.77) |
| Immunoregulatory interactions between a Lymphoid and a non-Lymphoid cell | 5 | 3 | **SELL** (-0.77), **FCGR3A** (-0.75), **B2M** (-0.42) |
| Platelet degranulation | 44 | 9 | **PPBP** (-1.89), **LGALS3BP** (-1.07), **HRG** (-1.05), **FGB** (-0.88), **F13A1** (-0.83), **ITIH3** (-0.83), **ECM1** (-0.71), **FGG** (-0.67), **SPARC** (-0.64), |
| Response to elevated platelet cytosolic Ca2+ | 44 | 9 | **PPBP** (-1.89), **LGALS3BP** (-1.07), **HRG** (-1.05), **FGB** (-0.88), F13A1 (-0.83), **ITIH3** (-0.83), **ECM1** (-0.71), **FGG** (-0.67), **SPARC** (-0.64), |
| Gluconeogenesis | 5 | 3 | **PGK1** (-0.58), **GAPDH** (-0.45), **PGAM2** (-0.42) |


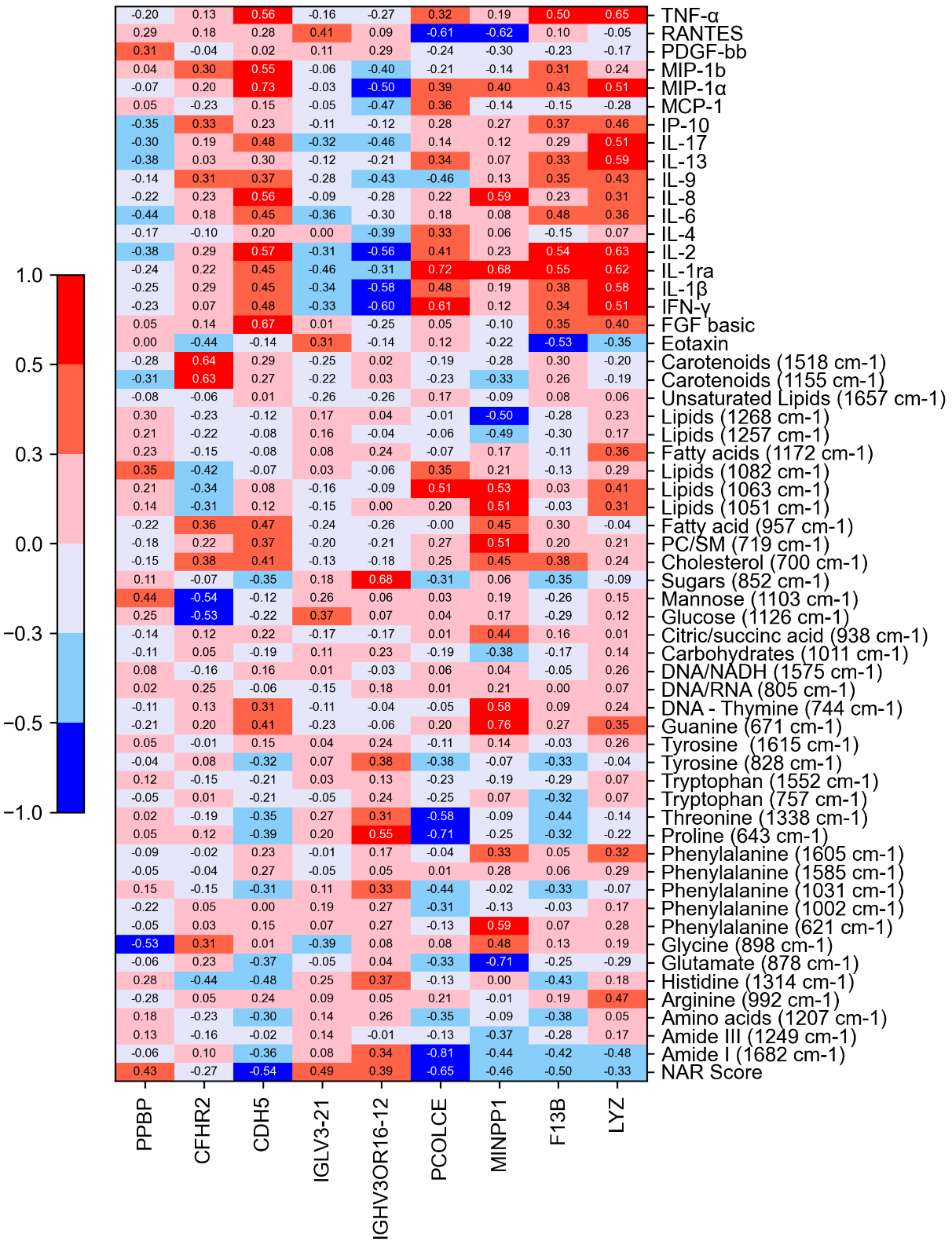


**Figure S14**. Annotated correlation heatmap of differentially expressed serum proteins to RS metabolites and serum signaling proteins. The differentially expressed proteins identified from LC-MS proteomics include platelet basic protein (PPBP), complement factor H related 2 (CFHR2), cadherin-5 (CDH5), immunoglobulin lambda variable 3-21 (IGLV3-21), immunoglobulin heavy variable 3/OR16-12 (IGHV3OR16-12), procollagen C-endopeptidase enhancer 1 (PCOLCE), multiple inositol polyphosphate phosphatase 1 (MINPP1), coagulation factor XIII (F13B) and lysozyme C (LYZ). Correlations were conducted with n = 8 complete responder and n = 10 poor responder LARC patients.
